# Development of a Triplex Real-Time PCR Method for the Simultaneous Detection of Porcine Circovirus 2, 3, and 4 in China Between 2023 and 2024

**DOI:** 10.3390/v17060777

**Published:** 2025-05-29

**Authors:** Yanhong Chen, Yi Lu, Dongfan Li, Ling Dong, Yang Zeng, Zhijing Mei, Ahmed H. Ghonaim, USAMA, Zhixian Yu, Shuo Zhang, Ping Bai, Wentao Li, Xuexiang Yu, Qigai He

**Affiliations:** 1 National Key Laboratory of Agricultural Microbiology, College of Veterinary Medicine, Huazhong Agricultural University, Wuhan 430070, China; chenyanhong@webmail.hzau.edu.cn (Y.C.); luyi0828_fire@webmail.hzau.edu.cn (Y.L.); lidongfan@webmail.hzau.edu.cn (D.L.); dong1001@webmail.hzau.edu.cn (L.D.); zy0802@webmail.hzau.edu.cn (Y.Z.); meizhijing@webmail.hzau.edu.cn (Z.M.); a.ghonaim@webmail.hzau.edu.cn (A.H.G.); usama7860@webmail.hzau.edu.cn (U.); wentao@mail.hzau.edu.cn (W.L.); 2 The Cooperative Innovation Center for Sustainable Pig Production, Huazhong Agricultural University, Wuhan 430070, China; 3 Desert Research Center, Cairo 11435, Egypt; 4 Yunnan Southwest Agriculture and Animal Husbandry Group Co., Ltd., Kunming 650000, China; 13786232775@163.com (Z.Y.); 13700603279@163.com (S.Z.); 18608717666@163.com (P.B.)

**Keywords:** porcine circovirus, triplex qPCR, genetic evolution analysis, genotype

## Abstract

Background: Porcine circovirus disease (PCVD), caused by porcine circovirus (PCV), is a significant swine disease characterized by porcine dermatitis, nephrotic syndrome, and reproductive disorders in sows. Given the overlapping clinical presentations of PCV2, PCV3, and PCV4, a rapid and accurate method for their differential detection is essential. Methods: In this study, specific primers and probes were designed based on the conserved regions of the *ORF1* genes of PCV2 and PCV4, as well as the *ORF2* gene of PCV3. Results: A TaqMan triple real-time PCR method was developed, demonstrating excellent specificity, sensitivity, and repeatability, with limits of detection (LODs) of 53.3 copies/µL, 12.0 copies/µL, and 13.8 copies/µL for PCV2, PCV3, and PCV4, respectively. Using this method, 500 clinical porcine tissue samples collected from 23 provinces across China between 2023 and 2024 were analyzed. The results showed detection rates of 75.20% (376/500) for PCV2, 17.60% (88/500) for PCV3, and 4.40% (22/500) for PCV4. The detection rate of triple coinfections involving PCV2, PCV3, and PCV4 was 0.80% (4/500). PCV2 consistently presented significantly higher positive detection rates across all growth stages, and its viral copy number was significantly greater than those of PCV3 and PCV4 (* *p* < 0.05). Forty PCV2 *ORF2* genes, fourteen PCV3 *ORF2* genes, and three PCV4 *ORF2* genes were identified. These included four PCV2a genotypes, thirty-five PCV2d genotypes, and one PCV2e genotypes; two PCV3a genotypes and six each of PCV3b and PCV3c genotypes; and two PCV4a genotypes and one of PCV4b genotype. Conclusions: The triple qPCR method established in this study provides a rapid, specific, and accurate approach for the detection and differentiation of PCV2, PCV3, and PCV4 genotypes.

## 1. Introduction

Porcine circovirus (PCV) is the primary pathogen responsible for porcine circovirus-associated disease (PCVAD), encompassing conditions including porcine dermatitis and nephropathy syndrome (PDNS), postweaning multisystemic wasting syndrome (PMWS), granulomatous enteritis, porcine respiratory disease complex, reproductive failure, and acute pulmonary edema [1,2]. Since the initial outbreak in Canada in 1997, PCVAD has spread to all pig-raising countries worldwide, posing a significant economic risk to the global pig farming industry.

PCV belongs to the family Circoviridae and the genus Circovirus and is recognized as the smallest known animal virus [3]. It is a nonenveloped, circular DNA virus with an icosahedral capsid structure, a diameter of 13–25 nm, and a genome size of approximately 1.7–2.0 kb [4]. According to the latest classification by the International Committee on Taxonomy of Viruses (ICTV), four recognized species of porcine circoviruses have been identified: PCV1, PCV2, PCV3, and PCV4 [4,5,6,7].

PCV1 was identified in 1974 during the cultivation of pig kidney cells (PK-15), and it is considered a nonpathogenic virus [8,9]. A novel PCV infection subsequently emerged in Canada, North America, and Europe [10,11]. Sequence analysis revealed substantial divergence from PCV1, leading to its classification as PCV2 [12]. To date, PCV2 has been classified into nine identified genotypes, designated PCV2a through PCV2i [13]. Among these, the primary genotypes PCV2a, PCV2b, and PCV2d are recognized globally, whereas the other genotypes exhibit more limited distributions [13,14]. In 2015, PCV3 was identified in sows exhibiting clinical symptoms similar to those of porcine dermatitis and nephropathy syndrome (PDNS), along with their mummified fetuses, via metagenomic sequencing [15]. Based on two amino acid mutations (A24V and R27K) in the Cap protein, PCV3 can be further divided into three distinct branches: PCV3a, PCV3b, and PCV3c [16]. In 2019, PCV4 was detected in pigs affected by respiratory disease, PDNS, and diarrhea through metagenomic sequencing [7]. While laboratory infection and clinical observations indicate that PCV1 is nonpathogenic, the other three PCVs, PCV2, PCV3, and PCV4, are established as pathogenic [17].

The clinical symptoms of PCV2, PCV3, and PCV4 are challenging to distinguish, complicating the control of porcine circovirus disease. Additionally, PCV infections in pig populations may arise from a single genotype or a combination of multiple genotypes [18,19]. Therefore, establishing a method that can simultaneously detect these three pathogens is essential. Multiplex quantitative real-time PCR (qPCR), also referred to as multiplex qPCR, is a rapid and sensitive detection technique that can identify multiple genes within a single reaction system. The genome sequence homology among PCV2, PCV3, and PCV4 ranges from 42.7% to 51.5% [7,15,20]. Owing to the relatively low genetic relatedness between these viruses, multiplex real-time fluorescence PCR technology can be effectively utilized to establish a detection method for different PCV genotypes.

In this study, we developed a TaqMan triplex qPCR detection method for the rapid identification of PCV2, PCV3, and PCV4. The results were compared with those of other detection methods to evaluate their accuracy. In addition, this method was preliminarily applied to 500 samples collected from various pig farms between 2023 and 2024. The aim of this study was to provide detection tools and relevant references for the clinical diagnosis of PCV and epidemiological investigations.

## 2. Materials and Methods

### 2.1. Primers and Probes

The GenBank database provides 78 complete porcine circovirus (PCV) genomes for analysis, comprising three subtypes: PCV2 (*n* = 30), PCV3 (*n* = 30), and PCV4 (*n* = 18). Subsequent multiple sequence alignment, performed using MEGA software, enabled the identification of subtype-specific conserved genomic regions. All primers and probes were designed using Primer 5.0 software, based on the sequences of the conserved regions of the *ORF1* gene of PCV2 (FJ598044.1) and PCV4 (MT311854.1) and the *ORF2* gene of PCV3 (MG546667.1). The qPCR amplification yielded the following fragment sizes: PCV2 (109 bp), PCV3 (130 bp), and PCV4 (105 bp). All primers and probes sequences synthesized by Beijing Tsingke Biotech Co., Ltd. (Beijing, China), are shown in Table 1.

### 2.2. Viruses and Clinical Samples

To evaluate the specificity of the triplex qPCR assay, we tested several viral pathogens, including porcine reproductive and respiratory syndrome (PRRSV, EU860248.1), porcine epidemic diarrhea virus (PEDV, KT021232.1), classical swine fever virus (CSFV, HM175885.1), porcine parvovirus (PPV, AY789532.1), and pseudorabies virus (PRV, AF306511.1). CSFV was obtained from commercial attenuated vaccines.

Between 2023 and 2024, a total of 500 clinical pig tissue samples were collected from 23 provinces. Approximately 1 cm diameter tissue samples were cut, placed in 2 mL Eppendorf tubes, cut into pieces, and supplemented with 800 µL of PBS buffer and sterilized glass beads. The mixture was placed into the tissue homogenizer and homogenized for 10 min. After 2–3 freeze–thaw cycles, the supernatant was collected and centrifuged to extract nucleic acid.

### 2.3. DNA/RNA Extraction and Reverse Transcription

Total nucleic acids (RNA/DNA) from PRRSV, PEDV, CSFV, PPV, and PRV were extracted from vaccine solutions or positive clinical samples via the Vazyme DNA/RNA Extraction Kit (Cat No. RM-201-02, Nanjing, China) following the manufacturer’s instructions. The extracted nucleic acids were eluted in 50 µL of nuclease-free water and stored at −80 °C. cDNA was synthesized via the use of HiScript^®^ III All-in-one RT SuperMix Perfect for RT-qPCR (Nanjing Vazyme Biotech Co., Ltd., Nanjing, China). The reverse transcription program included incubation at 50 °C for 15 min, followed by incubation at 85 °C for 5 s.

### 2.4. Construction of Recombinant Plasmids

The target fragments were amplified from PCV2 and PCV3 nucleic acids using qPCR primers. The PCR products were then cloned and inserted into a pMD18-T vector. To generate positive plasmids, the recombinant plasmids were transformed into DH5α competent cells. The bacterial cultures were shaken and incubated for 12–16 h at 37 °C. The plasmids were extracted using a HiSpeed Plasmid Mini Kit (Shanghai Qiagen Nanjing Biotech Co., Ltd., Shanghai, China) and sent for DNA sequencing. The recombinant standard plasmid constructs were confirmed by sequence analysis and named pMD18-PCV2 and pMD18-PCV3. The plasmid for pMD18-PCV4, was synthesized by Beijing Tsingke Biotech Co., Ltd. (Beijing, China). The ultraviolet absorbance of the standard plasmid constructs was measured at 260 nm and 280 nm, and their concentrations were calculated using the following formula: y (copies/µL) = (6.02 × 10^23^) × plasmid concentration ng/µL × 10^−9^ DNA)/(DNA length × 660).

### 2.5. Optimization of the Triplex qPCR Assay

The triplex real-time PCR assay was performed in a 20 µL reaction volume, which included 3 µL of three mixed standard plasmids, 10 µL of AceQ^®^ Universal U+ Probe Master Mix (Vazyme, Nanjing, China), 10 µL of each of the forward and reverse primers (100 nM, 200 nM, 300 nM, 400 nM, 500 nM, 600 nM, 700 nM, or 800 nM), each TaqMan probe (100 nM, 200 nM, 300 nM, 400 nM, or 500 nM), and nuclease-free water (up to 20 µL). The PCR assay was performed on a CFX96 Touch Real-Time PCR Detection System (Bio-Rad, Hercules, CA, USA) under the following conditions: initial incubation at 37 °C for 2 min and pre-denaturation at 95 °C for 30 s, followed by 40 cycles of 95 °C for 10–15 s and annealing at a temperature ranging from 56 °C to 60 °C for 30 s. The conditions yielding the lowest Ct values were selected for the optimal reaction.

### 2.6. Establishment of Standard Curves

To generate standard curves, a series of 10-fold dilutions (10^7^ copies/µL to 10^2^ copies/µL) were prepared for triple-time PCR assays using three standard plasmids, pMD18-PCV2, pMD18-PCV3, and pMD18-PCV4, as template DNA. Standard curves were generated based on the cycle threshold (Ct) values and the logarithmic (lg) copy numbers of the template DNA.

### 2.7. Specificity, Sensitivity, Repeatability, and Stability

To evaluate the analytical specificity of the developed triplex qPCR method, we utilized a range of viral pathogens, including PRRSV, PEDV, CSFV, PPV, and PRV. Each sample was tested in triplicate to validate the reliability of the results. This evaluation aimed to demonstrate the specificity of the triplex qPCR method in detecting various viral targets.

To evaluate the analytical sensitivity of the triplex qPCR method, three standard plasmids (pMD18-PCV2, pMD18-PCV3, and pMD18-PCV4) were diluted in 10-fold series down to 10^1^ copies/µL and amplified under the optimized reaction conditions to determine the detection limits across plasmid concentrations ranging from 10^7^ to 10^1^ copies/µL. To evaluate the limits of detection in different sample types, three mixed plasmids (concentration range 10^5^ to 10^1^ copies/µL) were mixed into swabs, lymphoid, anticoagulated blood, and sera separately to simulate clinical samples. These simulants were extracted and assayed under optimized reaction conditions.

For a reproducibility (intra-batch) and stability (inter-batch) assessment of the developed triplex qPCR, standard plasmids (pMD18-PCV2, pMD18-PCV3, and pMD18-PCV4) diluted to 10^7^ copies/µL, 10^5^ copies/µL, and 10^3^ copies/µL were analyzed, and the corresponding Ct values were recorded at different time points. The intra-batch and inter-batch coefficients of variation (CVs) of less than 5% indicate high repeatability and stability.

### 2.8. Comparison of the Detection Results of Different Methods

To evaluate the accuracy of the method in clinical samples, three mixed plasmids were spiked into 200 μL of serum (*n* = 10), 0.5 g of lung tissue (*n* = 10), and 0.5 g of lymphoid tissue (*n* = 10) at a concentration of 10^5^ copies/µL to simulate clinical samples (Table S1). Additionally, 30 mixed samples of clinical spleen tissue and lymph nodes were tested. Genomic DNA was extracted from these samples and analyzed using both the detection method developed in this study and the reference method [21,22]. The reference methods are singleplex real-time qPCR assays for PCV2, PCV3, and PCV4 genotypes, respectively. The results from both methods were compared via the kappa consistency test to assess their agreement.

### 2.9. Clinical Sample Detection

Between 2023 and 2024, a total of 500 clinical swine samples—each consisting of pooled organ mixtures (heart, liver, spleen, lungs, kidneys, lymph nodes, and aborted fetus) collected from individual pigs during necropsy—were obtained from 23 provinces of China (Table S2). These samples included 275 samples from asymptomatic pigs and 225 samples from symptomatic pigs exhibiting clinical signs such as diarrhea, emaciation, abortion, or respiratory and neurological disorders.

### 2.10. PCV2/PCV3/PCV4 Sequence Analyses

Primers were used to amplify the PCV2, PCV3, and PCV4 *ORF2* genes’ sequences, respectively. All primers’ sequences synthesized by Beijing Tsingke Biotech Co., Ltd. (Bei-jing, China) are shown in Table S3. We utilized MEGA6.0 and Megalign11.0 software to align the obtained sequences with the reference sequences and analyzed their genetic variability and phylogenetic relationships. The genomic sequences of the reference strains in this study are summarized in Table S4.

## 3. Results

### 3.1. Construction of Standard Plasmids

Using the primers PCV2F/R, PCV3F/R, and PCV4F/R, specific nucleic acid fragments were amplified, cloned, and inserted into the PMD-18T vector, which was subsequently transformed into DH5α cells. The copy numbers of the standard plasmids were determined to be 5.23 × 10^10^ copies/µL, 1.20 × 10^11^ copies/µL, and 1.38 × 10^11^ copies/µL for pMD18-PCV2, pMD18-PCV3, and pMD18-PCV4, respectively.

### 3.2. Optimization of Triplex qPCR Reaction Conditions

For triplex real-time qPCR system optimization, annealing temperatures of 56 °C, 57 °C, 58 °C, 59 °C, and 60 °C, final primer concentrations ranging from 100 nM to 800 nM, and final probe concentrations ranging from 100 nM to 500 nM were used. On the basis of the optimization results, the optimal annealing temperature was determined to be 58 °C, with final primer concentrations of 600 nM, 500 nM, and 600 nM for pMD18-PCV2, pMD18-PCV3, and pMD18-PCV4, respectively, and a final probe concentration of 400 nM for each target (Tables S5 and S6). Therefore, the final amplification system comprised 10 μL of AceQ^®^ Universal U+ Probe Master Mix, 600 nM PCV2 primer, 500 nM PCV3 primer, and 600 nM PCV4 primer, each probe at a final concentration of 400 nM, 3 μL of template, and nuclease-free water to a final volume of 20 μL. The reaction conditions were as follows: 37 °C for 2 min and pre-denaturation at 95 °C for 30 s, followed by 40 cycles of 95 °C for 10 s, and 58 °C for 30 s (Table 2).

### 3.3. Establishment of Standard Curves

**Figure 1:**
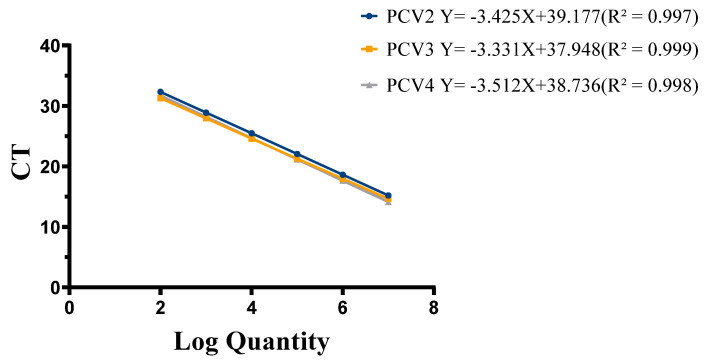
Establishment of standard curves. Standard curves for PCV2, PCV3, and PCV4. The amplification efficiency, slope, and correlation coefficient (R2) were 120.9%, −3.425, and 0.997 for pMD18-PCV2; 114.0%, −3.331, and 0.999 for pMD18-PCV3; and 93.96%, −3.512, and 0.998 for pMD18-PCV4.

To establish standard curves, the plasmids pMD18-PCV2, pMD18-PCV3, and pMD18-PCV4 were diluted in a 10-fold series from 1 × 10^7^ copies/µL to 1 × 10^2^ copies/µL and amplified via triplex real-time qPCR under the optimized reaction conditions and protocol. A standard curve was constructed using the cycle threshold (Ct) values as the vertical axis and the logarithm of the plasmid concentration as the horizontal axis. The amplification efficiency, slope, and correlation coefficient (R^2^) were 120.9%, −3.425, and 0.997 for PCV2; 114.0%, −3.331, and 0.999 for PCV3; and 93.96%, −3.512, and 0.998 for PCV4 (Figure 1).

### 3.4. Specificity, Sensitivity, Repeatability, and Stability

**Figure 2:**
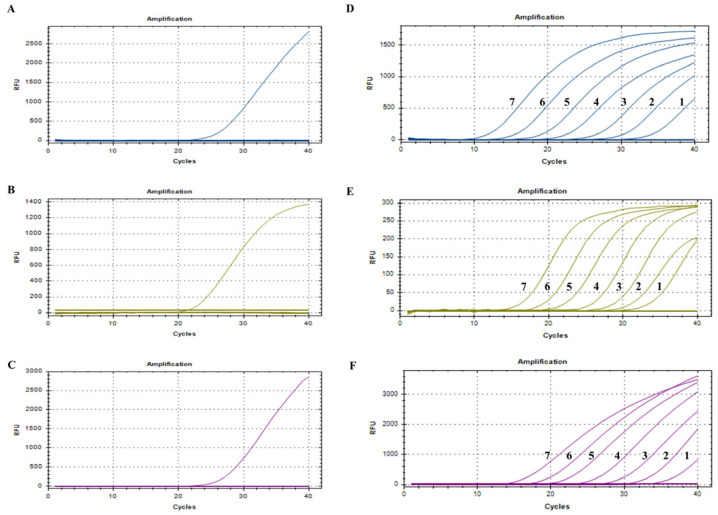
Detection limits and specificity of triplex qPCR methods: (**A**) PCV2-specific amplification curve (the result is blue). (**B**) PCV3-specific amplification curve (the result is yellow). (**C**) PCV4-specific amplification curve (the result is purple). (**D**) Detection limit for pMD18-PCV2. (**E**) Detection limit for pMD18-PCV3. (**F**) Detection limit for pMD18-PCV4.

The DNA or cDNA of PCV2, PCV3, PCV4, PRRSV, PEDV, CSFV, PPV, and PRV and RNase-free distilled water were used as templates for amplification. The results showed clear amplification curves for PCV2, PCV3, and PCV4, whereas no amplification curves were obtained for the PRRSV, PEDV, CSFV, PPV, and PRV. These findings indicate that the developed triplex qPCR method exhibits excellent specificity, with no positive signals detected for other viral pathogens (Figure 2A–C).

Triplex real-time qPCR was performed under optimal reaction conditions to amplify the target genes from three constructed plasmids (PCV2, PCV3, and PCV4) at seven concentrations ranging from 10^7^ copies/µL to 10^1^ copies/µL for the sensitivity test. As shown in Figure 2D–F, the limits of detection (LODs) were 53.3 copies/µL, 12.0 copies/µL, and 13.8 copies/µL for PCV2, PCV3, and PCV4, respectively (Figure 2D–F). The LODs of swabs, lymphoid, anticoagulated bloods, and sera simulated by plasmids were 1 × 10^1^ copies/µL, 1 × 10^2^ copies/µL, 1 × 10^1^ copies/µL, and 1 × 10^1^ copies/µL, respectively (Table S2), which indicated that the detection limits and amplification performance of the present triplex qPCR method are stable across different sample types.

To evaluate repeatability, three standard plasmid constructs at final reaction concentrations of 1 × 10^7^ copies/µL, 1 × 10^5^ copies/µL, and 1 × 10^3^ copies/µL were used as templates. The data indicated that the coefficients of variation (CVs) of the Ct values in the intra-assay and inter-assay reproducibility tests ranged from 0.43% to 0.63% and from 0.62% to 0.96%, respectively, demonstrating that the established method has excellent reproducibility (Table 3).

### 3.5. Comparison of the Detection Results of Different Methods

A total of 30 simulated clinical samples and 30 mixed spleen and lymph node tissues were analyzed via both the developed assay and reference methods. A comparison of the results from all 60 samples revealed that the assay developed demonstrated a high concordance rate with the reference methods, yielding a kappa coefficient of 0.97–1, indicating excellent agreement (Table 4).

### 3.6. Clinical Sample Detection

A total of 500 samples collected from 354 farms across 23 provinces were analyzed using the developed assay. The results revealed that PCV2 nucleic acid was present in all 23 provinces of China, with a farm-level detection rate of 75.14% (266/354, 95% CI: 70.64%~79.64%) and a sample-level detection rate of 75.20% (376/500, 95% CI: 71.41%~78.99%). PCV3 was detected at a farm-level rate of 17.80% (63/354, 95%CI: 13.81%~21.78%), and a sample-level rate of 17.60% (88/500, 95% CI: 14.26%~20.94%). Additionally, 22 samples tested positive for PCV4, resulting in a farm-level detection rate of 5.65% (20/354, 95% CI: 3.24%~8.05%), and a sample-level detection rate of 4.40% (22/500, 95% CI: 2.60%~6.20%) (Table 5 and Table S7).

**Figure 3:**
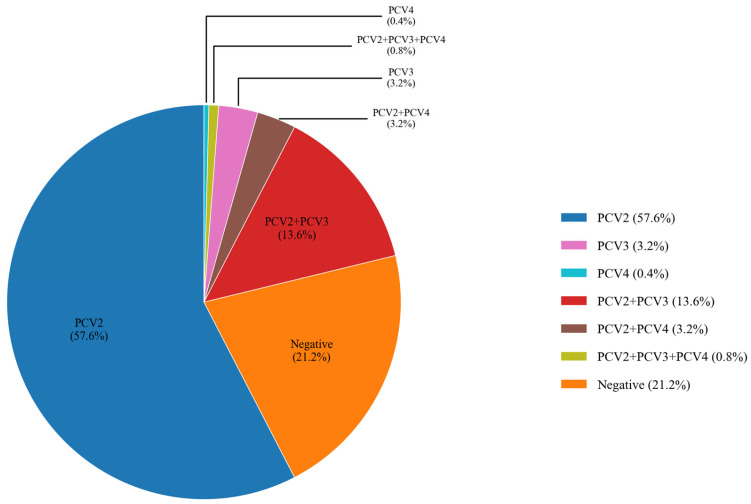
Analysis of the proportion for mixed infections of different genotypes. Different colors represent various types of infections. The results revealed that PCV2 had the highest detection rate as a single infection, while PCV3 and PCV4 were predominantly detected in coinfections with PCV2.

PCV is widely distributed on swine farms, with frequent clinical coinfections involving multiple genotypes. The results indicated that the detection rates for single infections of PCV2, PCV3, and PCV4 were 57.60% (288/500, 95% CI: 53.27%~61.93%), 3.20% (16/500, 95% CI: 1.66%~4.74%), and 0.40% (2/500, 95% CI: −0.15%~0.95%), respectively. Among coinfections, the most prevalent were PCV2 and PCV3, with a detection rate of 13.60% (68/500, 95% CI: 10.60%~16.60%). However, no cases of PCV3 and PCV4 coinfection were detected. The detection rate of triple coinfections involving PCV2, PCV3, and PCV4 was 0.80% (4/500, 95% CI: 0.02%~1.58%). The results revealed that in clinical settings, PCV3 and PCV4 are primarily detected in coinfections with PCV2 (Figure 3).

### 3.7. Detection of PCV2, PCV3, and PCV4 in Different Samples

**Figure 4:**
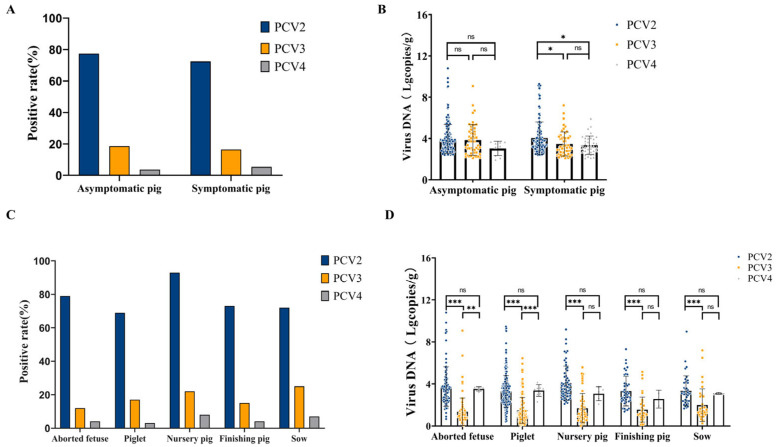
PCV2, PCV3, and PCV4 positive rates in asymptomatic and symptomatic pigs at different growth stages. (**A**) Positive rates and (**B**) viral copy numbers of PCV2, PCV3, and PCV4 in asymptomatic and symptomatic pigs. * Indicates statistically significant differences in the mean viral copy number in symptomatic pigs (* *p* < 0.05). (**C**) Positive rates and (**D**) viral copy numbers of PCV2, PCV3, and PCV4 in different growth stages of pigs. ** Indicates statistically significant differences in the mean PCV3 and PCV4 viral copy numbers in aborted fetuses stage.*** Indicates statistically significant differences in the mean PCV2 and PCV3 viral copy numbers in samples from all growth stages (*** *p* < 0.001).

The samples were further classified into asymptomatic group (275 samples) and symptomatic group (225 samples), and the positive detection rate of PCV2 was significantly higher than those of PCV3 and PCV4 in both groups. The positive detection rates for PCV2 in the asymptomatic and symptomatic groups were 77.45% (213/275, 95% CI: 72.52%~82.39%) and 72.44% (163/225, 95% CI: 66.61%~78.28%), respectively, whereas those for PCV3 were 18.55% (51/275, 95% CI: 13.95%~23.14%) and 16.44% (37/225, 95% CI: 11.60%~21.29%), respectively. PCV4 positivity was the lowest, at 3.64% (10/275, 95% CI: 1.42%~5.85%) in the asymptomatic group and 5.33% (12/225, 95% CI: 2.40%~8.27%) in the symptomatic group. In general, PCV2 has a relatively high prevalence in swine populations. Further quantitative analysis revealed that, in the symptomatic group, the mean viral copy number of PCV2 was significantly higher than that of PCV3 and PCV4 (* *p* < 0.05), suggesting that PCV2 has greater pathogenicity in clinical settings (Figure 4A,B).

Based on the growth stages of pigs, the samples were classified into five production stages: aborted fetuses, piglets (≤21 days old), nursery pigs (postweaning up to 70 days old), finishing pigs (70–180 days old), and sows. As shown in Figure 4C, the positive detection rate of PCV2 was consistently higher than that of PCV3 and PCV4 across all growth stages. Notably, nursery pigs and aborted fetuses presented the highest PCV2-positive rates, reaching 93.55% (58/62, 95% CI: 87.43%~99.66%) and 79.38% (77/97, 95% CI: 71.33%~87.43%), respectively. The detection rate of PCV3 was relatively high in sows (25.00%, 10/40, 95% CI: 11.58%~38.42%) and nursery pigs (22.58%, 14/62, 95% CI: 12.17%~32.99%), and PCV4 positivity was low in all growth stages, ranging from 3.13% (8/256, 95% CI: 0.99%~5.26%) to 8.06% (5/62, 95% CI: 1.29%~14.84%). These findings indicate that the prevalence of PCV4 is considerably lower than that of PCV2 and PCV3. Quantitative analysis further showed that the viral copy number of PCV2 was significantly higher than that of PCV3 (*** *p* < 0.001), and this difference was consistent across all stages of the samples, further suggesting that PCV2 has a higher pathogenic potential in pigs. The viral copy numbers of PCV3 and PCV4 did not differ significantly at most stages (Figure 4C,D).

### 3.8. PCV2/PCV3/PCV4 Genetic Characteristics

**Figure 5:**
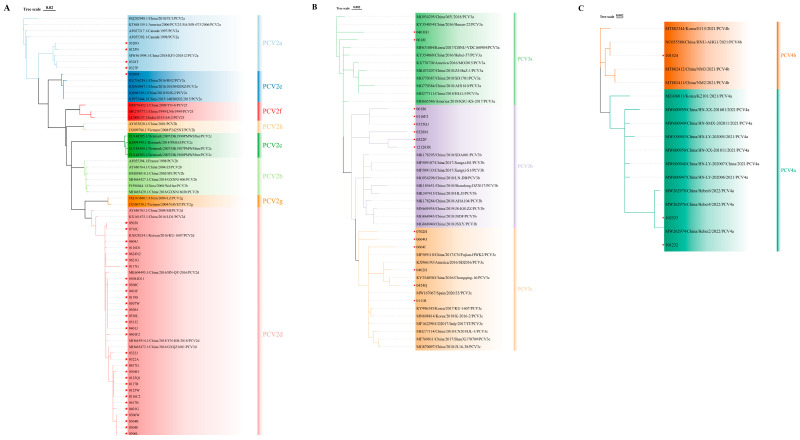
Phylogenetic tree of the *ORF2* gene of PCV2 and PCV3. (**A**) Phylogenetic analysis of the PCV2 *ORF2* gene. (**B**) Phylogenetic analysis of the PCV3 *ORF2* gene. (**C**) Phylogenetic analysis of the PCV4 *ORF2* gene. The phylogenetic tree was constructed using the neighbor-joining method in MEGA 6.0 software (p-distance model; 1000 bootstrap replicates). The red stars represent the virus strains obtained in this study. Different genotypes are indicated by distinct colors in the figures.

We amplified the full-length *ORF2* gene from the positive samples, obtaining forty PCV2 *ORF2* gene sequences, fourteen PCV3 *ORF2* gene sequences, and three PCV4 *ORF2* gene sequences. The genetic evolution of the PCV2 *ORF2* gene sequences obtained in this study was analyzed using MEGA 6.0 software and compared with the *ORF2* gene sequences of 32 PCV2 reference strains. Phylogenetic analysis identified three subtypes among the 40 sequenced strains: PCV2a, PCV2d, and PCV2e. Specifically, four strains were classified as PCV2a, thirty-five as PCV2d, and one as PCV2e. For the PCV3 *ORF2* gene sequences, we compared our 14 sequenced strains with those of 30 PCV3 reference strains. Phylogenetic analysis classified the 14 sequenced PCV3 strains into three subtypes: two of PCV3a genotypes, and six each of PCV3b and PCV3c genotypes. The analysis of the three PCV4 sequences against 14 reference strains identified two as PCV4a and one as PCV4b (Figure 5).

Nucleotide homology analysis was subsequently conducted via Megalign software. Nucleotide sequence analysis revealed that the PCV2 *ORF2* gene sequences from the 40 strains presented nucleotide homology ranging from 88.4% to 100.0%. Compared with those of the reference strain, their homologies ranged from 86.1% to 99.9%. The homology of the 14 PCV3 *ORF2* gene sequences ranged from 98.1% to 100.0%. Compared with those of the reference strain, the PCV3 *ORF2* gene sequences of the 14 strains presented nucleotide homology of 97.9% to 100.0%. PCV4 *ORF2* sequence homology of the three strains ranged from 97.8% to 100.0% compared with reference strain homology ranging from 97.7%~100.0% (Figures S1–S3).

## 4. Discussion

Porcine circovirus disease (PCVD) refers to a group of immunosuppressive diseases caused by porcine circoviruses, including PCV2, PCV3, and PCV4. PCVD is associated with various clinical syndromes, including postweaning multisystemic wasting syndrome (PMWS), porcine dermatitis and nephropathy syndrome (PDNS), porcine respiratory disease complex (PRDC), congenital tremors (CTs) in piglets, reproductive disorders, and granulomatous enteritis [7,23,24,25,26,27,28].

Globally, surveillance data from the U.S. Midwest between 2016 and 2018 revealed that the coinfection rate of PCV2 and PCV3 steadily increased from 3.4% in 2016 to 16.1% in 2018 [13]. The serology of clinically healthy pigs across nine European countries revealed positive rates of PCV2, PCV3, and PCV2/3 coinfection of 21%, 8%, and 3%, respectively, in finishing pigs [29]. Extensive surveillance data on PCV infections have also been reported in China [30]. Between 2015 and 2018, the reported infection rates of PCV2, PCV3, and PCV2/3 coinfection were 50.0%, 13.3%, and 6.78%, respectively [31]. Between 2018 and 2020, the PCV2/3 coinfection rate further increased to 19.7% [19]. Between 2020 and 2022, the infection rates of PCV2, PCV3, and PCV4 in Eastern China reached 31.03%, 30.09%, and 30.84%, respectively, with a coinfection rate of 28.22% [32]. Notably, as an emerged pathogen, PCV4 has been detected in both clinically healthy pig herds and those coinfected with PCV2 and PCV3 [33,34]. Multiple studies have confirmed the widespread global prevalence of PCV2, PCV3, and PCV4 coinfections, with a notable increasing trend in PCV2 and PCV3 coinfections. The emergence of new porcine circoviruses (PCVs), the widespread occurrence of mixed infections with different genotypes, the continuous evolution of circulating prevalent strains, and the incomplete development of commercial vaccines have significantly compromised pig farm productivity and posed substantial challenges for PCV prevention and control. To address these challenges, this study aims to establish a TaqMan multiplex qPCR detection method for the simultaneous identification and diagnosis of PCV2, PCV3, and PCV4 and to investigate their prevalence and molecular epidemiology in selected regions of China. Given that the homology range of the whole-genome sequences of PCV2, PCV3, and PCV4 is only 42.7–51.5% [15], primers and probes targeting the *ORF1* genes of PCV2 and PCV4 and the *ORF2* gene of PCV3 were designed through a comparative analysis of their whole-genome sequences. To optimize the reaction conditions, five annealing temperatures were tested and the concentrations of the primers and probes were optimized by checkerboard assay. After verification, this optimized system demonstrated the ability to detect PCV plasmids of PCV2, PCV3, and PCV4 with the detection limits of 53.3 copies/µL, 12.0 copies/µL, and 13.8 copies/µL, respectively. Therefore, the assay established in this study serves as a valuable tool for rapid and accurate diagnoses of different types of PCVs.

The triplex qPCR method established in this study was employed to analyze 500 samples collected from 354 farms across 23 provinces in China. PCV2 was detected in all 23 provinces, with prevalence rates ranging from 63.64% to 88.24%. In our survey, PCV3 detection rates were 23.81% in Northeast China and 20.99% in Southwest China, and PCV4 was detected at rates ranging from 2.94% to 9.52% across regions. Our data indicate that PCVs are widely distributed and highly prevalent in clinical production and pig breeding.

Interestingly, we observed that PCV2 and PCV3 positivity rates were both higher in the asymptomatic group than in the symptomatic group. This may be attributed to the distinct virological and immunological dynamics between these populations. PCV2 and PCV3 can establish persistent infections in swine, with asymptomatic individuals often harboring latent infections characterized by low viral loads and prolonged shedding without overt clinical signs [35]. In contrast, the activation of the host immune response in symptomatic pigs may transiently reduce detectable viral DNA in tissues, as robust immunity has been linked to diminished viral persistence [17]. The timing of sampling and immune dynamics may further influence these observations: symptomatic pigs are often sampled later in the course of infection, when host immune responses can reduce tissue viral loads, whereas asymptomatic pigs sampled without regard to clinical stage may include many animals in the early or peak viremia phase, when viral copy numbers are highest [2,23]. Furthermore, asymptomatic pigs may mount more effective innate or adaptive responses that constrain viral replication to subclinical levels [36]. This complex interplay underscores the need for integrated diagnostics combining viral quantification, coinfection profiling, and immune-status evaluation to fully elucidate PCV pathogenesis.

PCV2 is detected at high rates in different health statuses or growth stages, and its viral copy number remains high even in asymptomatic pigs. In contrast, the prevalence of PCV3 and PCV4 is relatively low, although a certain positive detection rate is observed in symptomatic pigs. This observation suggests that PCV2 may exhibit a broader or more consistent distribution in subclinical states. In symptomatic infections, PCV2 exhibited a significantly higher viral copy number compared to PCV3 and PCV4 (Figure 4B), indicating a greater replication capacity and suggesting enhanced pathogenic potential in clinically affected pigs. Its high viral copy number may exacerbate host immunosuppression and facilitate secondary infections.

Notably, this study was conducted as a passive surveillance investigation, with most clinical tissue samples collected from pigs exhibiting suspected or confirmed disease symptoms. Consequently, the detection rate reported in this study may differ from those reported in other investigations. The inherent selection bias in the sample collection process, which primarily targeted symptomatic pigs, may have led to either an overestimation or underestimation of the true prevalence of PCV within the overall pig population.

Epidemiological investigations revealed that PCV2a, PCV2b, and PCV2d exhibit a cyclical pattern of epidemic circulation on a global scale. Prior to 2003, PCV2a was the predominant genotype; however, it was gradually replaced by PCV2b as the prevalent genotype [37,38]. Since 2012, PCV2d has emerged as the primary prevalent genotype on pig farms worldwide [39]. The epidemiological trends of PCV2 in China largely align with the global situation, with PCV2d being the predominant circulating genotype [40].

In this study, phylogenetic analysis confirmed that PCV2d was the most prevalent strain at 87.50% (35/40). A comparison of the nucleotide sequences of the *ORF2* gene of PCV2 indicated that the nucleotide homology between the PCV2d strain identified in this study and the PCV2d reference strain ranged from 96.0% to 100.0%, whereas the amino acid sequence homology ranged from 91.8% to 100.0% (Figures S4 and S5). In contrast to PCV2, PCV3 has lower genetic diversity. Nucleotide sequence comparison of the *ORF2* gene with the PCV3 reference strain revealed homology ranging from 97.9% to 100.0%, whereas amino acid sequence homology ranged from 96.3% to 100.0% (Figures S2 and S6). Further genetic evolutionary analyses of PCV2 and PCV3 were performed to distinguish their genetic subtypes, which are critical for vaccine development and disease control. PCV4, an emerging circovirus, has low genomic homology with other PCVs. PCV4 has only 50.3%, 51.5%, and 43.2% homology with PCV1, PCV2, and PCV3, respectively [7]. Unlike other PCV genotypes, the PCV4 genome has high homology, suggesting that it is stable with little variation [41]. Given the low PCV4 positivity rate, the continuous surveillance of swine herds is necessary to accurately assess PCV4 prevalence.

In addition, PCVs can be detected in pigs coinfected with various swine pathogens, including PRRSV, PRV, PEDV, PPV, SIV, and CSFV [42,43,44,45,46]. Given the high prevalence of mixed infections in swine farms and their potential to exacerbate disease severity, the routine surveillance of pig farms is essential. Owing to the high mutation rate of the PCV2 genome and the existence of multiple PCV2 subtypes, PCV2 vaccines are continuously being optimized. Currently, there are no commercial vaccines available for PCV3 or PCV4 because these viruses exhibit considerable genetic and antigenic divergence. Consequently, it remains uncertain whether the PCV2 vaccine provides cross-protective effects against other PCV genotypes [4]. The regular monitoring of pig farms is imperative for tracking the epidemiological dynamics of circovirus infections and providing critical data for the development of effective prevention and control strategies. The findings of this study emphasize the need for continuous surveillance and effective control strategies within the swine industry. They also serve as a vital reference for understanding PCV epidemiology in China and for formulating targeted prevention and control measures.

## 5. Conclusions

In conclusion, this study established a clinically applicable detection and genotyping method for PCV. The established triplex qPCR assay enables rapid, multiplex detection of PCV2, PCV3, and PCV4 in a single reaction with high sensitivity and cost-effectiveness. The primers and probes exhibit high specificity without interference from off-target binding sites, and the assay’s performance on both laboratory-simulated samples and clinical specimens surpasses that of other fluorescence-based quantitative PCR methods. In addition, the findings of this study serve as a crucial reference for understanding the epidemiological characteristics of PCVs in China and provide valuable insights for developing targeted prevention and control measures.

## Figures and Tables

**Table 1:** Primers and probes.

Pathogen	Primer	Sequence (5′-3′)	Length (bp)	Gene
PCV2	PCV2-F	GGAGTCTGGTGACCGTTGC	109 bp	*ORF1*
	PCV2-R	TTCCAATCCCGCTTCTGCATT		
	PCV2-P	FAM-CCGCTCACTTTCAAAAGTTCAGCCA-BHQ1		
PCV3	PCV3-F	AGTGCTCCCCATTGAACG	130 bp	*ORF2*
	PCV3-R	GCCGTTACTTCACCCCCAA		
	PCV3-P	HEX-AGAGGCTTTGTCCTGGGTGAGC- BHQ1		
PCV4	PCV4-F	AAGCGCAGCGACCTTAAA	105 bp	*ORF1*
	PCV4-R	GCCACGCCCATACCTTAT		
	PCV4-P	VIC-GCCCGTGAGTTCCCGTCTGT-BHQ1		

**Table 2:** Optimized triplex qPCR system.

Reagent	Volume (μL)
PCV2-F (10 μM)	0.6 μL
PCV2-R (10 μM)	0.6 μL
PCV2-P (10 μM)	0.4 μL
PCV3-F (10 μM)	0.5 μL
PCV3-R (10 μM)	0.5 μL
PCV3-P (10 μM)	0.4 μL
PCV4-F (10 μM)	0.6 μL
PCV4-R (10 μM)	0.6 μL
PCV4-P (10 μM)	0.4 μL
Nucleic acids	3 μL
RNase-free distilled water	2.4 μL
AceQ^®^ Universal U+ Probe Master Mix	10 μL
Total	20 μL

**Table 3:** Repeatability and reproducibility analyses of the multiplex qPCR assay.

Plasmid Construct	Concentration (Copies/µL)	Intra-Assay Ct Values		Inter-Assay Ct Values	
		Mean ± SD	CV (%)	Mean ± SD	CV (%)
pMD18T-PCV2	5.23 × 10^7^	12.68 ± 0.08	0.63	12.73 ± 0.12	0.94
	5.23 × 10^5^	19.58 ± 0.11	0.56	19.62 ± 0.15	0.76
	5.23 × 10^3^	26.44 ± 0.13	0.49	26.48 ± 0.17	0.64
pMD18T-PCV3	1.20 × 10^7^	14.35 ± 0.07	0.49	14.38 ± 0.10	0.70
	1.20 × 10^5^	21.03 ± 0.10	0.48	21.07 ± 0.13	0.62
	1.20 × 10^3^	27.68 ± 0.15	0.54	27.74 ± 0.20	0.72
pMD18T-PCV4	1.38 × 10^7^	13.64 ± 0.06	0.44	13.66 ± 0.09	0.66
	1.38 × 10^5^	20.69 ± 0.09	0.43	20.73 ± 0.14	0.68
	1.38 × 10^3^	27.72 ± 0.14	0.50	27.77 ± 0.19	0.68

**Table 4:** Comparison of the consistency of the triple qPCR with the reference method.

Kappa Test = 1		Triple qPCR Method		Total
		+	−	
PCV2 Reference Method	+	48	0	48
	−	0	12	12
Total		48	12	60
Kappa Test = 1		Triple qPCR Method		Total
		+	−	
PCV3 Reference Method	+	42	0	42
	−	0	18	18
Total		42	18	60
Kappa Test = 0.9667		Triple qPCR Method		Total
		+	−	
PCV4 Reference Method	+	29	0	29
	−	1	30	31
Total		30	30	60

**Table 5:** Summary of PCV detection in 500 pig samples.

		PCV2		PCV3		PCV4	
Region	Number of Samples	Positive Samples	Positivity Rate	Positive Samples	Positivity Rate	Positive Samples	Positivity Rate
North China	117	89	76.07% (89/117)	22	18.80% (22/117)	5	4.27% (5/117)
Northeast China	21	16	76.19% (16/21)	5	23.81% (5/21)	2	9.52% (2/21)
Eastern China	91	76	83.52% (76/91)	17	18.68% (17/91)	3	3.30% (3/91)
Central China	77	49	63.64% (49/77)	11	14.29% (11/77)	4	5.19% (4/77)
South China	79	63	79.75% (63/79)	10	12.66% (10/79)	4	5.06% (4/79)
Southwest China	81	53	65.43% (53/81)	17	20.99% (17/81)	3	3.70% (3/81)
Northwest China	34	30	88.24% (30/34)	6	17.65% (6/34)	1	2.94% (1/34)
Total	500	376	75.20% (376/500)	88	17.60% (88/500)	22	4.40% (22/500)

## Data Availability

The data presented in this study are available in Supplementary Materials here.

## Supplementary Materials

The following supporting information can be downloaded at https://www.mdpi.com/article/10.3390/v17060777/s1. Table S1: Comparison of results between the reference method and the triple qPCR method. Table S2: Limits of detection in different sample types. Table S3: Amplification sequence of PCV2/PCV3 *ORF2* genes. Table S4: Information of PCV2 and PCV3 reference strains. Table S5: Optimization of primer concentration and probe concentration for triplex qPCR assays. Table S6: Optimization of the annealing temperature of the triplex qPCR assay. Table S7: Statistics of PCV positive rate of samples in different regions. Figure S1: Nucleotide homology analysis of the PCV2 *ORF2* gene. Figure S2: Nucleotide homology analysis of the PCV3 *ORF2* gene. Figure S3: Nucleotide homology analysis of the PCV4 *ORF2* gene. Figure S4: Nucleotide homology analysis of the PCV2d *ORF2* gene. Figure S5: Amino acid homology analysis of the PCV2d *ORF2* gene. Figure S6: Amino acid homology analysis of the PCV3 *ORF2* gene.
