# Supplementary material for: Development of a Triplex Real-Time PCR Method for the Simultaneous Detection of Porcine Circovirus 2, 3, and 4 in China Between 2023 and 2024
Source: Viruses. 2025 May 29;17(6):777. doi: 10.3390/v17060777 (PMC12197758; doi:10.3390/v17060777)
Supplement: Supplementary file 1 [file viruses-17-00777-s001.zip › viruses-3613614-supplementary.pdf]

Supplementary Materials

Supplementary Table S1 Comparison of results between the reference method and the triple qPCR method.

Numbers	Sample Types	The triple qPCR method established in this study			Reference qPCR methods		
		PCV2	PCV3	PCV4	PCV2	PCV3	PCV4
S-1	Serum	+	+	+	+	+	+
S-2	Serum	+	+	+	+	+	+
S-3	Serum	+	+	+	+	+	+
S-4	Serum	+	+	+	+	+	+
S-5	Serum	+	+	+	+	+	+
S-6	Serum	+	+	+	+	+	+
S-7	Serum	+	+	+	+	+	+
S-8	Serum	+	+	+	+	+	+
S-9	Serum	+	+	+	+	+	-
S-10	Serum	+	+	+	+	+	+
LU-1	Lung tissue sample	+	+	+	+	+	+
LU-2	Lung tissue sample	+	+	+	+	+	+
LU-3	Lung tissue sample	+	+	+	+	+	+
LU-4	Lung tissue sample	+	+	+	+	+	+
LU-5	Lung tissue sample	+	+	+	+	+	+
LU-6	Lung tissue sample	+	+	+	+	+	+
LU-7	Lung tissue sample	+	+	+	+	+	+
LU-8	Lung tissue sample	+	+	+	+	+	+
LU-9	Lung tissue sample	+	+	+	+	+	+
LU-10	Lung tissue sample	+	+	+	+	+	+
LY-1	Lymphoid tissue sample	+	+	+	+	+	+
LY-2	Lymphoid tissue sample	+	+	+	+	+	+
LY-3	Lymphoid tissue sample	+	+	+	+	+	+
LY-4	Lymphoid tissue sample	+	+	+	+	+	+
LY-5	Lymphoid tissue sample	+	+	+	+	+	+
LY-6	Lymphoid tissue sample	+	+	+	+	+	+
LY-7	Lymphoid tissue sample	+	+	+	+	+	+
LY-8	Lymphoid tissue sample	+	+	+	+	+	+
LY-9	Lymphoid tissue sample	+	+	+	+	+	+
LY-10	Lymphoid tissue sample	+	+	+	+	+	+
M-1	Mixed spleen and lymph node tissues	+	+	-	+	+	-
M-2	Mixed spleen and lymph node tissues	-	+	-	-	+	-
M-3	Mixed spleen and lymph node tissues	+	-	-	+	-	-
M-4	Mixed spleen and lymph node tissues	+	-	-	+	-	-
M-5	Mixed spleen and lymph node tissues	-	-	-	-	-	-
M-6	Mixed spleen and lymph node tissues	+	+	-	+	+	-
M-7	Mixed spleen and lymph node tissues	+	-	-	+	-	-
M-8	Mixed spleen and lymph node tissues	+	-	-	+	-	-

M-9	Mixed spleen and lymph node tissues	+	-	-	+	-	-
M-10	Mixed spleen and lymph node tissues	+	+	-	+	+	-
M-11	Mixed spleen and lymph node tissues	-	-	-	-	-	-
M-12	Mixed spleen and lymph node tissues	-	-	-	-	-	-
M-13	Mixed spleen and lymph node tissues	+	+	-	+	+	-
M-14	Mixed spleen and lymph node tissues	-	-	-	-	-	-
M-15	Mixed spleen and lymph node tissues	+	+	-	+	+	-
M-16	Mixed spleen and lymph node tissues	-	+	-	-	+	-
M-17	Mixed spleen and lymph node tissues	+	+	-	+	+	-
M-18	Mixed spleen and lymph node tissues	+	+	-	+	+	-
M-19	Mixed spleen and lymph node tissues	-	-	-	-	-	-
M-20	Mixed spleen and lymph node tissues	+	-	-	+	-	-
M-21	Mixed spleen and lymph node tissues	+	-	-	+	-	-
M-22	Mixed spleen and lymph node tissues	-	-	-	-	-	-
M-23	Mixed spleen and lymph node tissues	+	-	-	+	-	-
M-24	Mixed spleen and lymph node tissues	+	+	-	+	+	-
M-25	Mixed spleen and lymph node tissues	-	-	-	-	-	-
M-26	Mixed spleen and lymph node tissues	+	+	-	+	+	-
M-27	Mixed spleen and lymph node tissues	-	-	-	-	-	-
M-29	Mixed spleen and lymph node tissues	+	-	-	+	-	-
M-30	Mixed spleen and lymph node tissues	-	+	-	-	+	-
	Positive control	+	+	+	+	+	+
	Negative control	-	-	-	-	-	-

Supplementary Table S2 Limits of detection in different sample types.

Sample Type	PCV2 LODs (copies/μL)	PCV2 Mean ± SD	PCV3 LODs (copies/μL)	PCV3 Mean ± SD	PCV4 LODs (copies/μL)	PCV4 Mean ± SD
Swabs	1×10^1	30.56 ± 0.25	1×10^1	34.58 ± 0.25	1×10^1	34.71 ± 0.13
Lymphoid	1×10^2	29.14 ± 0.31	1×10^2	30.29 ± 0.21	1×10^2	29.47 ±0.28
Anticoagulated bloods	1×10^1	32.06 ± 0.20	1×10^2	30.57 ± 0.27	1×10^1	29.21 ± 0.30
Sera	1×10^1	32.56 ± 0.22	1×10^1	31.02 ± 0.24	1×10^1	30.61 ± 0.20

Supplementary Table S3 Amplification sequence of PCV2/PCV3 ORF2 genes.

Primer	Sequence (5’-3’)	Length (bp)
PCV2ORF2-F	GAACAATCCACGGAGGAAGGGGGCC	918 bp
PCV2ORF2-R	CTTCTTGCTGGGCATGTTGCTGC	

PCV3ORF2-F	CATGCGAGGGCGTTTACCTGTGCC	963 bp
PCV3ORF2-R	AAAACTCAGTTATCCAATCCCCGT	
PCV4ORF2-F	CAGACTTTATTTGTGTTATCACTT	972 bp
PCV4ORF2-R	AGGTAAGGAATTCTTTAAGTTTCT	

Supplementary Table S4 Information of PCV2 and PCV3 reference strains.

Strain name	Genotype	GenBank Login Number	Origins	Separatist year
PCV2	PCV2a	AF055392.1	Canada	1998
PCV2	PCV2a	AF027217.1	Canada	1997
TC1	PCV2a	HQ202948	China	2010
10JS-2	PCV2e	JQ806749	China	2010
KF3-201812	PCV2a	MW561994	China	2018
MN-073	PCV2a	KT868159.1	America	2006
WuHan-PCV2	PCV2b	FJ598044.1	China	2008
GXNN1603b	PCV2b	MH465429	China	2016
BY	PCV2b	HM038018	China	2005
GXNN1406	PCV2b	MH465427	China	2014
France-PCV2	PCV2b	AF055394.1	France	1998
ZJ	PCV2b	AY686764.1	China	2004
DK1987PMWSfree	PCV2c	EU148504.1	Denmark	2007
DK1990PMWSfree	PCV2c	EU148505.1	Denmark	2007
PM163	PCV2c	KJ094599.1	Denmark	2014
DK1980PMWSfree	PCV2c	EU148503	Denmark	2007
GXQZ1601	PCV2d	MH465472	China	2016
HN-QY-2016	PCV2d	MK604495	China	2016
YN-HH-2018	PCV2d	MH645914	China	2018
KU-1607	PCV2d	KX828234	Korean	2016
LD1	PCV2d	KX161675.1	China	2016
SH	PCV2d	AY686763.1	China	2004
20150420XZ	PCV2e	KX960947.1	China	2016
RN2	PCV2e	KU756238.1	China	2016
14HB0202	PCV2e	KP975444.1	China	2015
LN6	PCV2f	MF278777	China	1999
AS-2	PCV2f	LC008137	India	2013
YN-8	PCV2f	HM776452	China	2009
P2425NT	PCV2h	JX099786	Vietnam	2008
LZ	PCV2g	DQ363860	China	2006
NAVET	PCV2g	JX506730	Vietnam	2004
PCV2	PCV2h	AY035820	China	2001
MY	PCV3a	MG934295	China	2018
Hunan-22	PCV3a	KY354054	China	2016
CBNU-VDC160984	PCV3a	MF631804	Korea	2017

Hubei-57	PCV3a	KY354068	China	2016
2018HLG-5	PCV3a	MH277111	China	2018
AH1610	PCV3a	MG770384	China	2018
SD1701	PCV3a	MG770387	China	2018
MO2015	PCV3a	KX778720	America	2016
ZJ-HuZ-1	PCV3a	MK033207	China	2018
KSU-KS-2017	PCV3a	MH603546	America	2018
B1	PCV3b	MF589107	China	2017
Jiangxi-S1	PCV3b	MF589133	China	2017
SDA001	PCV3b	MK178295	China	2018
Shandong-DZ2017	PCV3b	MK185651	China	2018
HLJ3	PCV3b	MK347415	China	2018
LN/DB	PCV3b	MG934298	China	2018
AHA104	PCV3b	MK178284	China	2018
JS-K01ZZ	PCV3b	MN605934	China	2019
JSDF	PCV3b	MG868943	China	2018
JSXY	PCV3b	MG868940	China	2018
SD2016	PCV3c	KX966193	America	2016
Chongqing-16	PCV3c	KY354050	China	2016
IT/CO2017	PCV3c	MF162298	Italy	2017
35	PCV3c	MW167067	Spain	2020
KU-1607	PCV3c	KY996343	Korea	2017
K-2016-2	PCV3c	MN698814	Korea	2019
CN2018JL-3	PCV3c	MH277114	China	2018
ShanXi170709	PCV3c	MF769811	China	2017
Fujian-HWK2	PCV3c	MF589110	China	2017
JL16-38	PCV3c	MG870097	China	2018
HN-LY-202005	PCV4a	MW538943	China	2021
HN-SMX-202011	PCV4a	MW600949	China	2021
HN-XX-201811	PCV4a	MW600950	China	2021
HN-LY-202007	PCV4a	MW600948	China	2021
HN-LY-202006	PCV4a	MW600947	China	2021
HN-XX-201601	PCV4a	MW600959	China	2021
Hebei4	PCV4a	MW262976	China	2022
Hebei6	PCV4a	MW262978	China	2022
Hebei2	PCV4a	MW262974	China	2022
Hebei5	PCV4a	MW262977	China	2022
MZ436811/Korea/K2101/2021/PCV4a	PCV4a	MZ436811	Korea	2021
NM2	PCV4b	MT882411	China	2021
NM3	PCV4b	MT882412	China	2021
HNU-AHG1	PCV4b	NC055580	China	2021
FJ	PCV4b	MT721742	China	2021
E115	PCV4b	MT882344	Korea	2021

Supplementary Table S5 Optimization of primer concentration and probe concentration for triplex qPCR assays.

	PCV2(FAM)				
Probe concentration(nM)	Primer concentration(nM)				
	100	200	300	400	500
100	30.38±0.12	32.04±0.27	35.51±0.20	30.04±0.16	31.99±0.13
200	28.22±0.22	28.28±0.22	28.26±0.20	27.63±0.17	28.27±0.16
300	28.09±0.25	28.06±0.33	28.13±0.23	27.48±0.23	28.29±0.21
400	27.61±0.30	27.70±0.21	27.50±0.21	27.00±0.07	27.72±0.39
500	27.40±0.35	27.83±0.38	27.56±0.37	27.26±0.36	27.60±0.31
700	27.25±0.40	27.50±0.42	27.36±0.41	26.71±0.38	27.51±0.98
600	27.12±0.15	27.21±0.39	27.18±0.39	26.35±0.28	27.48±0.34
800	26.98±0.19	26.88±0.20	27.10±0.36	27.02±0.37	27.39±0.27
	PCV3(VIC)				
Probe concentration(nM)	Primer concentration(nM)				
	100	200	300	400	500
100	26.29±0.05	26.38±0.12	26.36±0.30	26.17±0.45	25.93±1.10
200	26.21±0.18	25.64±0.72	25.47±0.98	25.64±0.20	25.37±0.85
300	26.06±0.33	25.62±1.25	25.47±0.50	25.64±0.65	25.25±0.15
400	26.54±0.90	25.68±0.55	25.48±0.40	25.52±0.02	25.24±0.07
500	26.52±1.50	25.60±0.28	25.29±0.07	25.22±0.66	25.34±0.60
600	26.30±0.80	25.55±1.00	25.37±1.75	25.30±0.38	25.32±0.09
700	26.59±0.14	25.93±0.23	25.46±0.01	25.38±0.25	25.40±0.48
800	26.21±0.78	25.70±0.04	25.43±0.35	25.39±0.95	25.32±1.20
	PCV4(CY5)				
Probe concentration(nM)	Primer concentration(nM)				
	100	200	300	400	500
100	29.53±0.02	32.00±0.10	30.37±0.25	31.88±0.40	29.61±1.20
200	27.85±0.22	27.24±0.68	27.58±0.95	27.47±0.18	27.29±0.75
300	26.33±0.30	26.02±0.22	26.27±1.35	26.21±0.55	26.14±0.60
400	25.62±0.50	25.60±0.35	25.39±0.03	25.73±0.05	25.47±0.14
500	25.29±0.85	25.40±1.70	25.29±0.27	25.26±0.08	25.16±0.72
600	25.31±1.10	25.56±1.90	25.33±0.37	25.13±0.09	25.31±0.62
700	25.28±0.80	25.19±0.12	25.22±0.21	25.35±0.01	25.18±0.28
800	25.31±0.22	25.30±0.32	25.47±0.98	25.42±1.15	25.52±0.45

Supplementary Table S6 Optimization of the annealing temperature of the triplex qPCR assay.

Annealing temperature	Template		
	PCV2	PCV3	PCV4
60°C	29.16± 0.30	27.55±0.35	29.6±0.28

59°C	29.64±0.22	27.39±0.29	30.05±0.25
58°C	29.16±0.12	27.23±0.18	29.5±0.14
57°C	29.86±0.18	27.24±0.24	29.53±0.20
56°C	30.01±0.27	27.41±0.33	29.38±0.31

Supplementary Table S7 Statistics of PCV positive rate of samples in different regions.

Sample source	Number of pig farms	Number of samples	PCV2 positive field	PCV2 positive samples	PCV3 positive field	PCV3 positive samples	PCV4 positive field	PCV4 positive samples
Anhui	12	17	9	14	5	10	0	0
Fujian	5	8	4	7	1	1	0	0
Gansu	3	4	2	3	0	0	0	0
Guangdong	28	36	23	31	4	7	4	4
Guangxi	23	43	17	32	2	3	0	0
Guizhou	8	10	6	8	1	1	0	0
Hebei	24	34	18	26	2	3	1	1
Henan	39	60	35	52	12	17	4	4
Heilongjiang	4	5	3	4	1	1	0	0
Hubei	18	27	9	17	6	10	1	1
Hunan	15	23	11	15	1	1	1	1
Jiangsu	8	11	7	10	0	0	2	2
Jiangxi	24	27	15	17	0	0	1	2
Liaoning	6	10	4	7	0	0	0	0
Neimenggu	2	6	2	5	2	4	1	2
Shandong	27	42	25	35	4	5	0	0
Shanxi	18	23	11	11	2	2	0	0
Shaanxi	20	28	19	25	4	6	1	1
Sichuan	42	49	25	29	13	14	1	1
Xinjiang	2	2	2	2	0	0	0	0
Yunnan	3	4	2	2	0	0	1	1
Zhejiang	10	13	8	10	1	1	1	1
Chongqing	13	18	9	14	2	2	1	1
Total	354	500	266	376	63	88	20	22

Supplementary Figure S1. Nucleotide homology analysis of the PCV2 ORF2 gene.

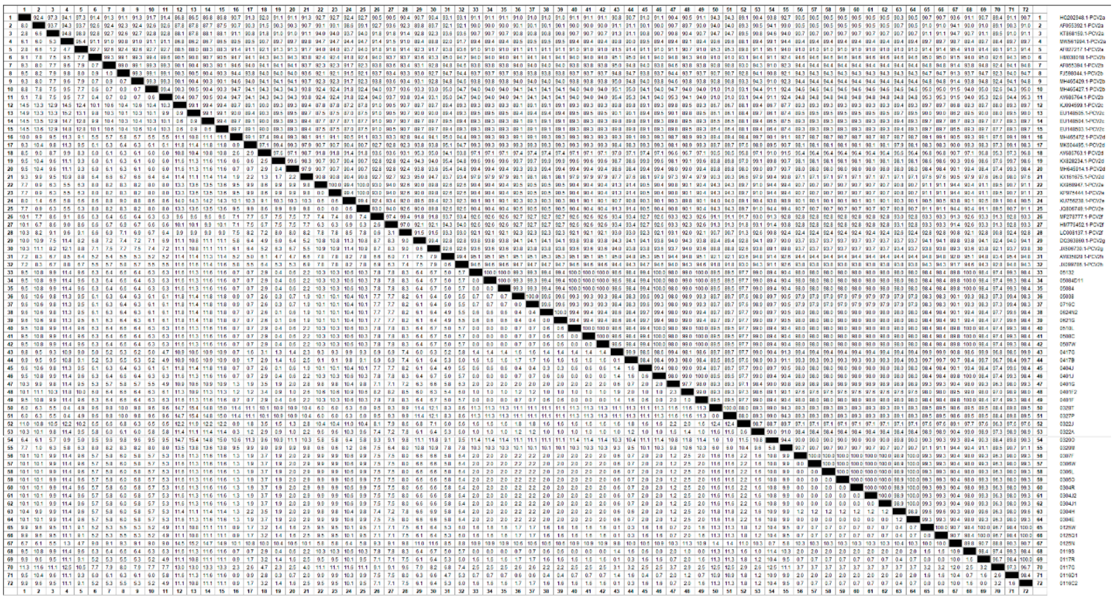

Supplementary Figure S2. Nucleotide homology analysis of the PCV3 ORF2 gene.

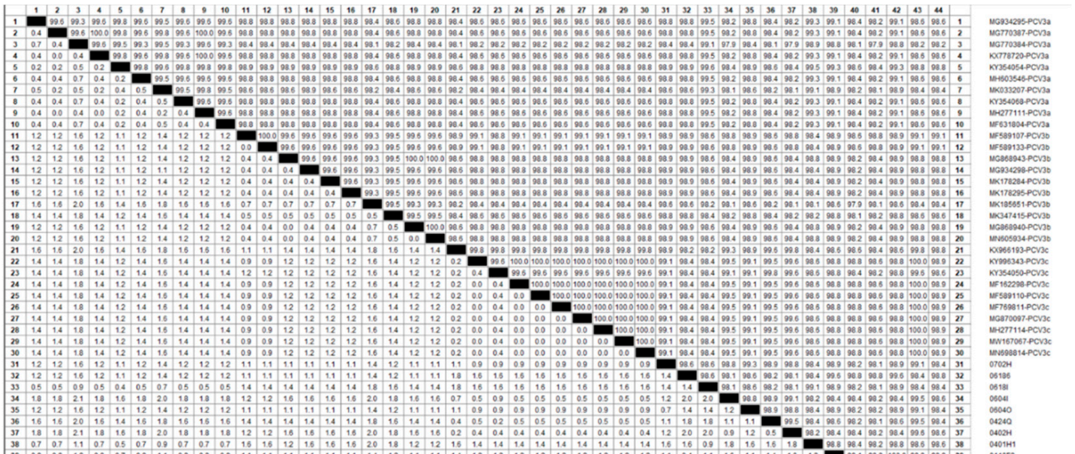

Supplementary Figure S3. Nucleotide homology analysis of the PCV4 ORF2 gene.

	1	2	3	4	5	6	7	8	9	10	11	12	13	14	15	16	17		
1		98.5	99.0	98.8	98.8	98.8	98.7	98.0	98.1	97.8	98.7	98.8	98.8	98.7	98.8	97.8	97.8	1	MZ436811-PCV4a
2	1.5		99.6	99.4	99.4	99.4	99.3	98.5	98.7	98.4	98.4	98.5	98.5	98.4	98.5	98.4	98.4	2	MW600959-PCV4a
3	1.0	0.4		99.9	99.9	99.9	99.7	99.0	99.1	98.8	98.8	99.0	99.0	98.8	99.0	98.8	98.8	3	MW600950-PCV4a
4	1.2	0.6	0.1		99.7	99.7	99.6	98.8	99.0	98.7	98.7	98.8	98.8	98.7	98.8	98.7	98.7	4	MW600949-PCV4a
5	1.2	0.6	0.1	0.3		99.7	99.6	98.8	99.0	98.7	98.7	98.8	98.8	98.7	98.8	98.7	98.7	5	MW600948-PCV4a
6	1.2	0.6	0.1	0.3	0.3		99.6	98.8	99.0	98.7	98.7	98.8	98.8	98.7	98.8	98.7	98.7	6	MW600947-PCV4a
7	1.3	0.7	0.3	0.4	0.4	0.4		98.7	98.8	98.5	98.5	98.7	98.7	98.5	98.7	98.5	98.5	7	MW538943-PCV4a
8	2.1	1.5	1.0	1.2	1.2	1.2	1.3		99.9	99.6	97.8	98.3	98.0	97.8	98.0	99.6	99.6	8	MW262978-PCV4a
9	1.9	1.3	0.9	1.0	1.0	1.0	1.2	0.1		99.7	98.0	98.4	98.1	98.0	98.1	99.7	99.7	9	MW262976-PCV4a
10	2.2	1.6	1.2	1.3	1.3	1.3	1.5	0.4	0.3		97.7	98.1	97.8	97.7	97.8	100.0	100.0	10	MW262974-PCV4a
11	1.3	1.6	1.2	1.3	1.3	1.3	1.5	2.2	2.1	2.4		99.3	99.6	100.0	99.6	97.7	97.7	11	MT882412-PCV4b
12	1.2	1.5	1.0	1.2	1.2	1.2	1.3	1.8	1.6	1.9	0.7		99.4	99.3	99.4	98.1	98.1	12	MT882344-PCV4b
13	1.2	1.5	1.0	1.2	1.2	1.2	1.3	2.1	1.9	2.2	0.4	0.6		99.6	100.0	97.8	97.8	13	NC055580-PCV4b
14	1.3	1.6	1.2	1.3	1.3	1.3	1.5	2.2	2.1	2.4	0.0	0.7	0.4		99.6	97.7	97.7	14	MT882411-PCV4b
15	1.2	1.5	1.0	1.2	1.2	1.2	1.3	2.1	1.9	2.2	0.4	0.6	0.0	0.4		97.8	97.8	15	101524
16	2.2	1.6	1.2	1.3	1.3	1.3	1.5	0.4	0.3	0.0	2.4	1.9	2.2	2.4	2.2		100.0	16	102523
17	2.2	1.6	1.2	1.3	1.3	1.3	1.5	0.4	0.3	0.0	2.4	1.9	2.2	2.4	2.2	0.0		17	101232
	1	2	3	4	5	6	7	8	9	10	11	12	13	14	15	16	17		

Supplementary Figure S4. Nucleotide homology analysis of the PCV2d ORF2 gene.

	1	2	3	4	5	6	7	8	9	10	11	12	13	14	15	16	17	18	19	20	21	22	23	24	25	26	27	28	29	30	31	32	33	34	35	36	37	38	39	40	41		
1		99.1	97.4	99.4	99.3	98.1	99.3	99.3	99.3	99.1	99.1	99.3	99.3	99.3	99.3	99.3	99.3	99.1	99.3	99.3	98.7	98.9	99.3	98.6	99.7	98.7	98.7	98.7	98.7	98.7	98.7	98.7	98.7	98.7	99.1	99.1	99.3	99.1	97.6	99.1	99.1	1	MH465472.1-PCV2d
2	0.9		97.1	99.4	99.3	97.9	99.3	99.3	99.3	99.3	99.3	99.3	99.3	99.3	99.3	99.3	98.4	98.3	99.3	99.3	98.1	98.9	99.3	97.7	98.9	98.1	98.1	98.1	98.1	98.1	98.1	98.1	97.9	98.1	98.3	98.3	99.3	98.3	97.3	99.1	98.3	2	MK604495.1-PCV2d
3	2.6	2.9		97.6	97.1	98.7	97.1	97.1	97.1	97.4	97.4	97.4	97.4	97.1	97.1	97.1	97.0	97.1	97.4	97.1	96.6	96.7	97.1	96.0	97.1	96.4	96.4	96.4	96.4	96.4	96.4	96.4	96.6	96.4	96.9	96.9	97.1	96.9	95.3	97.3	96.9	3	AY686763.1-PCV2d
4	0.6	0.6	2.5		99.6	98.3	99.6	99.6	99.6	99.7	99.7	99.9	99.9	99.6	99.6	99.6	98.7	98.6	99.9	99.6	98.1	99.1	99.6	98.0	99.1	98.1	98.1	98.1	98.1	98.1	98.1	98.1	98.1	98.1	98.6	98.6	99.6	98.6	97.6	99.7	98.6	4	KX828234.1-PCV2d
5	0.7	0.7	2.9	0.4		97.9	99.4	99.4	99.4	99.3	99.3	99.4	99.4	99.4	99.4	99.4	98.6	98.4	99.4	99.4	98.0	99.0	99.4	98.1	99.0	98.0	98.0	98.0	98.0	98.0	98.0	98.0	98.0	98.0	98.4	98.4	99.4	98.4	97.4	99.3	98.4	5	MH645914.1-PCV2d
6	1.9	2.2	1.3	1.7	2.2		97.9	97.9	97.9	98.1	98.1	98.1	98.1	97.9	97.9	97.9	97.7	97.6	98.1	97.9	97.3	97.4	97.9	96.7	97.9	97.1	97.1	97.1	97.1	97.1	97.1	97.1	97.1	97.1	97.6	97.6	97.9	97.6	96.0	98.0	97.6	6	KX161675.1-PCV2d
7	0.7	0.7	2.9	0.4	0.6	2.2		100.0	100.0	99.3	99.3	99.4	99.4	100.0	100.0	100.0	98.6	98.4	99.4	100.0	98.0	99.0	100.0	97.9	99.0	98.0	98.0	98.0	98.0	98.0	98.0	98.0	98.0	98.0	98.4	98.4	100.0	98.4	97.4	99.3	98.4	7	05132
8	0.7	0.7	2.9	0.4	0.6	2.2	0.0		100.0	99.3	99.3	99.4	99.4	100.0	100.0	100.0	98.6	98.4	99.4	100.0	98.0	99.0	100.0	97.9	99.0	98.0	98.0	98.0	98.0	98.0	98.0	98.0	98.0	98.0	98.4	98.4	100.0	98.4	97.4	99.3	98.4	8	05084D11
9	0.7	0.7	2.9	0.4	0.6	2.2	0.0	0.0		99.3	99.3	99.4	99.4	100.0	100.0	100.0	98.6	98.4	99.4	100.0	98.0	99.0	100.0	97.9	99.0	98.0	98.0	98.0	98.0	98.0	98.0	98.0	98.0	98.0	98.4	98.4	100.0	98.4	97.4	99.3	98.4	9	05084
10	0.9	0.7	2.6	0.3	0.7	1.9	0.7	0.7	0.7		100.0	99.6	99.6	99.3	99.3	99.3	98.4	98.3	99.6	99.3	98.0	98.9	99.3	97.7	98.9	97.9	97.9	97.9	97.9	97.9	97.9	97.9	97.9	97.9	98.3	98.3	99.3	98.3	97.3	99.4	98.3	10	05038
11	0.9	0.7	2.6	0.3	0.7	1.9	0.7	0.7	0.7	0.0		99.6	99.6	99.3	99.3	99.3	98.4	98.3	99.6	99.3	98.0	98.9	99.3	97.7	98.9	97.9	97.9	97.9	97.9	97.9	97.9	97.9	97.9	97.9	98.3	98.3	99.3	98.3	97.3	99.4	98.3	11	0716C
12	0.7	0.7	2.6	0.1	0.6	1.9	0.6	0.6	0.6	0.4	0.4		100.0	99.4	99.4	99.4	98.6	98.4	99.7	99.4	98.0	99.0	99.4	97.9	99.0	98.0	98.0	98.0	98.0	98.0	98.0	98.0	98.0	98.0	98.4	98.4	99.4	98.4	97.7	99.6	98.4	12	0624N2
13	0.7	0.7	2.6	0.1	0.6	1.9	0.6	0.6	0.6	0.4	0.4	0.0		99.4	99.4	99.4	98.6	98.4	99.7	99.4	98.0	99.0	99.4	97.9	99.0	98.0	98.0	98.0	98.0	98.0	98.0	98.0	98.0	98.0	98.4	98.4	99.4	98.4	97.7	99.6	98.4	13	0621G
14	0.7	0.7	2.9	0.4	0.6	2.2	0.0	0.0	0.0	0.7	0.7	0.6	0.6		100.0	100.0	98.6	98.4	99.4	100.0	98.0	99.0	100.0	97.9	99.0	98.0	98.0	98.0	98.0	98.0	98.0	98.0	98.0	98.0	98.4	98.4	100.0	98.4	97.4	99.3	98.4	14	0510L
15	0.7	0.7	2.9	0.4	0.6	2.2	0.0	0.0	0.0	0.7	0.7	0.6	0.6	0.0		100.0	98.6	98.4	99.4	100.0	98.0	99.0	100.0	97.9	99.0	98.0	98.0	98.0	98.0	98.0	98.0	98.0	98.0	98.0	98.4	98.4	100.0	98.4	97.4	99.3	98.4	15	0508C
16	0.7	0.7	2.9	0.4	0.6	2.2	0.0	0.0	0.0	0.7	0.7	0.6	0.6	0.0	0.0		98.6	98.4	99.4	100.0	98.0	99.0	100.0	97.9	99.0	98.0	98.0	98.0	98.0	98.0	98.0	98.0	98.0	98.0	98.4	98.4	100.0	98.4	97.4	99.3	98.4	16	0507W
17	0.7	1.6	3.1	1.3	1.4	2.3	1.4	1.4	1.4	1.6	1.6	1.4	1.4	1.4	1.4	1.4		99.9	98.6	98.6	99.4	98.1	98.6	97.9	99.0	99.4	99.4	99.4	99.4	99.4	99.4	99.4	99.4	99.4	99.9	99.9	98.6	99.9	96.9	98.6	99.9	17	0417G
18	0.9	1.7	2.9	1.4	1.6	2.5	1.6	1.6	1.6	1.7	1.7	1.6	1.6	1.6	1.6	1.6	0.1		98.4	98.4	99.3	98.0	98.4	97.7	98.9	99.3	99.3	99.3	99.3	99.3	99.3	99.3	99.4	99.3	99.7	99.7	98.4	99.7	96.7	98.4	99.7	18	0417B
19	0.7	0.7	2.6	0.1	0.6	1.9	0.6	0.6	0.6	0.4	0.4	0.3	0.3	0.6	0.6	0.6	1.4	1.6		99.4	98.0	99.0	99.4	97.9	99.0	98.0	98.0	98.0	98.0	98.0	98.0	98.0	98.0	98.0	98.4	98.4	99.4	98.4	97.4	99.6	98.4	19	0404J
20	0.7	0.7	2.9	0.4	0.6	2.2	0.0	0.0	0.0	0.7	0.7	0.6	0.6	0.0	0.0	0.0	1.4	1.6	0.6		98.0	99.0	100.0	97.9	99.0	98.0	98.0	98.0	98.0	98.0	98.0	98.0	98.0	98.0	98.4	98.4	100.0	98.4	97.4	99.3	98.4	20	0401J
21	1.3	1.9	3.5	1.9	2.0	2.8	2.0	2.0	2.0	2.0	2.0	2.0	2.0	2.0	2.0	2.0	0.6	0.7	2.0	2.0		97.7	98.0	97.3	98.4	98.9	98.9	98.9	98.9	98.9	98.9	98.9	98.9	98.9	99.3	99.3	98.0	99.3	96.3	98.0	99.3	21	0401G
22	1.2	1.2	3.4	0.9	1.0	2.6	1.0	1.0	1.0	1.2	1.2	1.0	1.0	1.0	1.0	1.0	1.9	2.0	1.0	1.0	2.3		99.0	97.4	98.6	97.6	97.6	97.6	97.6	97.6	97.6	97.6	97.6	97.6	98.0	98.0	99.0	98.0	97.0	98.9	98.0	22	0401F2
23	0.7	0.7	2.9	0.4	0.6	2.2	0.0	0.0	0.0	0.7	0.7	0.6	0.6	0.0	0.0	0.0	1.4	1.6	0.6	0.0	2.0	1.0		97.9	99.0	98.0	98.0	98.0	98.0	98.0	98.0	98.0	98.0	98.0	98.4	98.4	100.0	98.4	97.4	99.3	98.4	23	0401F
24	1.4	2.3	4.1	2.0	1.9	3.4	2.2	2.2	2.2	2.3	2.3	2.2	2.2	2.2	2.2	2.2	2.2	2.3	2.2	2.2	2.8	2.6	2.2		98.9	97.3	97.3	97.3	97.3	97.3	97.3	97.3	97.3	97.3	97.7	97.7	97.9	97.7	96.4	97.7	97.7	24	0322J
25	0.3	1.2	2.9	0.9	1.0	2.2	1.0	1.0	1.0	1.2	1.2	1.0	1.0	1.0	1.0	1.0	1.0	1.2	1.0	1.0	1.6	1.4	1.0	1.2		98.4	98.4	98.4	98.4	98.4	98.4	98.4	98.4	98.4	98.9	98.9	99.0	98.9	97.3	98.9	98.9	25	0322A
26	1.3	1.9	3.7	1.9	2.0	2.9	2.0	2.0	2.0	2.2	2.2	2.0	2.0	2.0	2.0	2.0	0.6	0.7	2.0	2.0	1.2	2.5	2.0	2.8	1.6		100.0	100.0	100.0	100.0	100.0	100.0	98.9	100.0	99.3	99.3	98.0	99.3	96.3	98.0	99.3	26	0307F
27	1.3	1.9	3.7	1.9	2.0	2.9	2.0	2.0	2.0	2.2	2.2	2.0	2.0	2.0	2.0	2.0	0.6	0.7	2.0	2.0	1.2	2.5	2.0	2.8	1.6	0.0		100.0	100.0	100.0	100.0	100.0	98.9	100.0	99.3	99.3	98.0	99.3	96.3	98.0	99.3	27	0306W
28	1.3	1.9	3.7	1.9	2.0	2.9	2.0	2.0	2.0	2.2	2.2	2.0	2.0	2.0	2.0	2.0	0.6	0.7	2.0	2.0	1.2	2.5	2.0	2.8	1.6	0.0	0.0		100.0	100.0	100.0	100.0	98.9	100.0	99.3	99.3	98.0	99.3	96.3	98.0	99.3	28	0306L
29	1.3	1.9	3.7	1.9	2.0	2.9	2.0	2.0	2.0	2.2	2.2	2.0	2.0	2.0	2.0	2.0	0.6	0.7	2.0	2.0	1.2	2.5	2.0	2.8	1.6	0.0	0.0	0.0		100.0	100.0	100.0	98.9	100.0	99.3	99.3	98.0	99.3	96.3	98.0	99.3	29	0305O
30	1.3	1.9	3.7	1.9	2.0	2.9	2.0	2.0	2.0	2.2	2.2	2.0	2.0	2.0	2.0	2.0	0.6	0.7	2.0	2.0	1.2	2.5	2.0	2.8	1.6	0.0	0.0	0.0	0.0		100.0	100.0	98.9	100.0	99.3	99.3	98.0	99.3	96.3	98.0	99.3	30	0304R
31	1.3	1.9	3.7	1.9	2.0	2.9	2.0	2.0	2.0	2.2	2.2	2.0	2.0	2.0	2.0	2.0	0.6	0.7	2.0	2.0	1.2	2.5	2.0	2.8	1.6	0.0	0.0	0.0	0.0	0.0		100.0	98.9	100.0	99.3	99.3	98.0	99.3	96.3	98.0	99.3	31	0304J2
32	1.3	1.9	3.7	1.9	2.0	2.9	2.0	2.0	2.0	2.2	2.2	2.0	2.0	2.0	2.0	2.0	0.6	0.7	2.0	2.0	1.2	2.5	2.0	2.8	1.6	0.0	0.0	0.0	0.0	0.0	0.0		98.9	100.0	99.3	99.3	98.0	99.3	96.3	98.0	99.3	32	0304J1
33	1.3	2.2	3.5	1.9	2.0	2.9	2.0	2.0	2.0	2.2	2.2	2.0	2.0	2.0	2.0	2.0	0.6	0.6	2.0	2.0	1.2	2.5	2.0	2.8	1.6	1.2	1.2	1.2	1.2	1.2	1.2	1.2		98.9	99.6	99.6	98.0	99.6	96.3	98.0	99.6	33	0304H
34	1.3	1.9	3.7	1.9	2.0	2.9	2.0	2.0	2.0	2.2	2.2	2.0	2.0	2.0	2.0	2.0	0.6	0.7	2.0	2.0	1.2	2.5	2.0	2.8	1.6	0.0	0.0	0.0	0.0	0.0	0.0	0.0	1.2		99.3	99.3	98.0	99.3	96.3	98.0	99.3	34	0304E
35	0.9	1.7	3.2	1.4	1.6	2.5	1.6	1.6	1.6	1.7	1.7	1.6	1.6	1.6	1.6	1.6	0.1	0.3	1.6	1.6	0.7	2.0	1.6	2.3	1.2	0.7	0.7	0.7	0.7	0.7	0.7	0.7	0.4	0.7		100.0	98.4	100.0	96.7	98.4	100.0	35	0125W
36	0.9	1.7	3.2	1.4	1.6	2.5	1.6	1.6	1.6	1.7	1.7	1.6	1.6	1.6	1.6	1.6	0.1	0.3	1.6	1.6	0.7	2.0	1.6	2.3	1.2	0.7	0.7	0.7	0.7	0.7	0.7	0.7	0.4	0.7	0.0		98.4	100.0	96.7	98.4	100.0	36	0125Q1
37	0.7	0.7	2.9	0.4	0.6	2.2	0.0	0.0	0.0	0.7	0.7	0.6	0.6	0.0	0.0	0.0	1.4	1.6	0.6	0.0	2.0	1.0	0.0	2.2	1.0	2.0	2.0	2.0	2.0	2.0	2.0	2.0	2.0	2.0	1.6	1.6		98.4	97.4	99.3	98.4	37	0119S
38	0.9	1.7	3.2	1.4	1.6	2.5	1.6	1.6	1.6	1.7	1.7	1.6	1.6	1.6	1.6	1.6	0.1	0.3	1.6	1.6	0.7	2.0	1.6	2.3	1.2	0.7	0.7	0.7	0.7	0.7	0.7	0.7	0.4	0.7	0.0	0.0	1.6		96.7	98.4	100.0	38	0117R
39	2.5	2.8	4.9	2.5	2.6	4.1	2.6	2.6	2.6	2.8	2.8	2.3	2.3	2.6	2.6	2.6	3.2	3.4	2.6	2.6	3.8	3.1	2.6	3.7	2.8	3.8	3.8	3.8	3.8	3.8	3.8	3.8	3.8	3.8	3.4	3.4	2.6	3.4		97.3	96.7	39	0117G
40	0.9	0.9	2.8	0.3	0.7	2.0	0.7	0.7	0.7	0.6	0.6	0.4	0.4	0.7	0.7	0.7	1.4	1.6	0.4	0.7	2.0	1.2	0.7	2.3	1.2	2.0	2.0	2.0	2.0	2.0	2.0	2.0	2.0	2.0	1.6	1.6	0.7	1.6	2.8		98.4	40	0116D1
41	0.9	1.7	3.2	1.4	1.6	2.5	1.6	1.6	1.6	1.7	1.7	1.6	1.6	1.6	1.6	1.6	0.1	0.3	1.6	1.6	0.7	2.0	1.6	2.3	1.2	0.7	0.7	0.7	0.7	0.7	0.7	0.7	0.4	0.7	0.0	0.0	1.6	0.0	3.4	1.6		41	0116C2
	1	2	3	4	5	6	7	8	9	10	11	12	13	14	15	16	17	18	19	20	21	22	23	24	25	26	27	28	29	30	31	32	33	34	35	36	37	38	39	40	41		

Supplementary Figure S5. Amino acid homology analysis of the PCV2d ORF2 gene.

	1	2	3	4	5	6	7	8	9	10	11	12	13	14	15	16	17	18	19	20	21	22	23	24	25	26	27	28	29	30	31	32	33	34	35	36	37	38	39	40	41		
1		98.7	96.1	98.7	99.1	97.4	98.7	98.7	98.7	97.9	97.9	98.7	98.7	98.7	98.7	98.7	98.7	98.7	98.7	98.7	98.3	98.7	98.7	96.6	99.6	98.3	98.3	98.3	98.3	98.3	98.3	98.3	97.9	98.3	98.7	98.7	98.7	98.7	94.8	97.9	98.7	1	MH465472.1-PCV2d
2	1.3		95.7	99.1	99.6	97.0	99.1	99.1	99.1	98.3	98.3	99.1	99.1	99.1	99.1	99.1	97.4	97.4	99.1	99.1	97.0	99.1	99.1	95.3	98.3	97.0	97.0	97.0	97.0	97.0	97.0	97.0	96.6	97.0	97.4	97.4	99.1	97.4	94.8	98.3	97.4	2	MK604495.1-PCV2d
3	4.0	4.4		96.6	96.1	98.7	95.7	95.7	95.7	96.6	96.6	96.6	96.6	95.7	95.7	95.7	95.7	95.7	96.6	95.7	95.3	95.7	95.7	92.7	95.7	95.3	95.3	95.3	95.3	95.3	95.3	95.3	94.8	95.3	95.7	95.7	95.7	95.7	91.8	95.7	95.7	3	AY686763.1-PCV2d
4	1.3	0.9	3.5		99.6	97.9	99.1	99.1	99.1	99.1	99.1	100.0	100.0	99.1	99.1	99.1	97.4	97.4	100.0	99.1	97.0	99.1	99.1	95.3	98.3	97.0	97.0	97.0	97.0	97.0	97.0	97.0	96.6	97.0	97.4	97.4	99.1	97.4	95.3	99.1	97.4	4	KX828234.1-PCV2d
5	0.9	0.4	4.0	0.4		97.4	99.6	99.6	99.6	98.7	98.7	99.6	99.6	99.6	99.6	99.6	97.9	97.9	99.6	99.6	97.4	99.6	99.6	95.7	98.7	97.4	97.4	97.4	97.4	97.4	97.4	97.4	97.0	97.4	97.9	97.9	99.6	97.9	95.3	98.7	97.9	5	MH645914.1-PCV2d
6	2.6	3.1	1.3	2.2	2.6		97.0	97.0	97.0	97.9	97.9	97.9	97.9	97.0	97.0	97.0	97.0	97.0	97.9	97.0	96.6	97.0	97.0	94.0	97.0	96.6	96.6	96.6	96.6	96.6	96.6	96.6	96.1	96.6	97.0	97.0	97.0	97.0	93.1	97.0	97.0	6	KX161675.1-PCV2d
7	1.3	0.9	4.4	0.9	0.4	3.1		100.0	100.0	98.3	98.3	99.1	99.1	100.0	100.0	100.0	97.4	97.4	99.1	100.0	97.0	99.1	100.0	95.3	98.3	97.0	97.0	97.0	97.0	97.0	97.0	97.0	96.6	97.0	97.4	97.4	100.0	97.4	94.8	98.3	97.4	7	05132
8	1.3	0.9	4.4	0.9	0.4	3.1	0.0		100.0	98.3	98.3	99.1	99.1	100.0	100.0	100.0	97.4	97.4	99.1	100.0	97.0	99.1	100.0	95.3	98.3	97.0	97.0	97.0	97.0	97.0	97.0	97.0	96.6	97.0	97.4	97.4	100.0	97.4	94.8	98.3	97.4	8	05084D11
9	1.3	0.9	4.4	0.9	0.4	3.1	0.0	0.0		98.3	98.3	99.1	99.1	100.0	100.0	100.0	97.4	97.4	99.1	100.0	97.0	99.1	100.0	95.3	98.3	97.0	97.0	97.0	97.0	97.0	97.0	97.0	96.6	97.0	97.4	97.4	100.0	97.4	94.8	98.3	97.4	9	05084
10	2.2	1.7	3.5	0.9	1.3	2.2	1.7	1.7	1.7		100.0	99.1	99.1	98.3	98.3	98.3	96.6	96.6	99.1	98.3	96.1	98.3	98.3	94.4	97.4	96.1	96.1	96.1	96.1	96.1	96.1	96.1	95.7	96.1	96.6	96.6	98.3	96.6	94.4	98.3	96.6	10	05038
11	2.2	1.7	3.5	0.9	1.3	2.2	1.7	1.7	1.7	0.0		99.1	99.1	98.3	98.3	98.3	96.6	96.6	99.1	98.3	96.1	98.3	98.3	94.4	97.4	96.1	96.1	96.1	96.1	96.1	96.1	96.1	95.7	96.1	96.6	96.6	98.3	96.6	94.4	98.3	96.6	11	0716C
12	1.3	0.9	3.5	0.0	0.4	2.2	0.9	0.9	0.9	0.9	0.9		100.0	99.1	99.1	99.1	97.4	97.4	100.0	99.1	97.0	99.1	99.1	95.3	98.3	97.0	97.0	97.0	97.0	97.0	97.0	97.0	96.6	97.0	97.4	97.4	99.1	97.4	95.3	99.1	97.4	12	0624N2
13	1.3	0.9	3.5	0.0	0.4	2.2	0.9	0.9	0.9	0.9	0.9	0.0		99.1	99.1	99.1	97.4	97.4	100.0	99.1	97.0	99.1	99.1	95.3	98.3	97.0	97.0	97.0	97.0	97.0	97.0	97.0	96.6	97.0	97.4	97.4	99.1	97.4	95.3	99.1	97.4	13	0621G
14	1.3	0.9	4.4	0.9	0.4	3.1	0.0	0.0	0.0	1.7	1.7	0.9	0.9		100.0	100.0	97.4	97.4	99.1	100.0	97.0	99.1	100.0	95.3	98.3	97.0	97.0	97.0	97.0	97.0	97.0	97.0	96.6	97.0	97.4	97.4	100.0	97.4	94.8	98.3	97.4	14	0510L
15	1.3	0.9	4.4	0.9	0.4	3.1	0.0	0.0	0.0	1.7	1.7	0.9	0.9	0.0		100.0	97.4	97.4	99.1	100.0	97.0	99.1	100.0	95.3	98.3	97.0	97.0	97.0	97.0	97.0	97.0	97.0	96.6	97.0	97.4	97.4	100.0	97.4	94.8	98.3	97.4	15	0508C
16	1.3	0.9	4.4	0.9	0.4	3.1	0.0	0.0	0.0	1.7	1.7	0.9	0.9	0.0	0.0		97.4	97.4	99.1	100.0	97.0	99.1	100.0	95.3	98.3	97.0	97.0	97.0	97.0	97.0	97.0	97.0	96.6	97.0	97.4	97.4	100.0	97.4	94.8	98.3	97.4	16	0507W
17	1.3	2.6	4.4	2.6	2.2	3.1	2.6	2.6	2.6	3.5	3.5	2.6	2.6	2.6	2.6	2.6		100.0	97.4	97.4	99.6	97.4	97.4	95.3	98.3	99.6	99.6	99.6	99.6	99.6	99.6	99.6	99.1	99.6	100.0	100.0	97.4	100.0	93.6	97.0	100.0	17	0417G
18	1.3	2.6	4.4	2.6	2.2	3.1	2.6	2.6	2.6	3.5	3.5	2.6	2.6	2.6	2.6	2.6	0.0		97.4	97.4	99.6	97.4	97.4	95.3	98.3	99.6	99.6	99.6	99.6	99.6	99.6	99.6	99.1	99.6	100.0	100.0	97.4	100.0	93.6	97.0	100.0	18	0417B
19	1.3	0.9	3.5	0.0	0.4	2.2	0.9	0.9	0.9	0.9	0.9	0.0	0.0	0.9	0.9	0.9	2.6	2.6		99.1	97.0	99.1	99.1	95.3	98.3	97.0	97.0	97.0	97.0	97.0	97.0	97.0	96.6	97.0	97.4	97.4	99.1	97.4	95.3	99.1	97.4	19	0404J
20	1.3	0.9	4.4	0.9	0.4	3.1	0.0	0.0	0.0	1.7	1.7	0.9	0.9	0.0	0.0	0.0	2.6	2.6	0.9		97.0	99.1	100.0	95.3	98.3	97.0	97.0	97.0	97.0	97.0	97.0	97.0	96.6	97.0	97.4	97.4	100.0	97.4	94.8	98.3	97.4	20	0401J
21	1.7	3.1	4.9	3.1	2.6	3.5	3.1	3.1	3.1	4.0	4.0	3.1	3.1	3.1	3.1	3.1	0.4	0.4	3.1	3.1		97.0	97.0	94.8	97.9	99.1	99.1	99.1	99.1	99.1	99.1	99.1	98.7	99.1	99.6	99.6	97.0	99.6	93.1	96.6	99.6	21	0401G
22	1.3	0.9	4.4	0.9	0.4	3.1	0.9	0.9	0.9	1.7	1.7	0.9	0.9	0.9	0.9	0.9	2.6	2.6	0.9	0.9	3.1		99.1	95.3	98.3	97.0	97.0	97.0	97.0	97.0	97.0	97.0	96.6	97.0	97.4	97.4	99.1	97.4	94.8	98.3	97.4	22	0401F2
23	1.3	0.9	4.4	0.9	0.4	3.1	0.0	0.0	0.0	1.7	1.7	0.9	0.9	0.0	0.0	0.0	2.6	2.6	0.9	0.0	3.1	0.9		95.3	98.3	97.0	97.0	97.0	97.0	97.0	97.0	97.0	96.6	97.0	97.4	97.4	100.0	97.4	94.8	98.3	97.4	23	0401F
24	3.5	4.9	7.7	4.9	4.4	6.3	4.9	4.9	4.9	5.8	5.8	4.9	4.9	4.9	4.9	4.9	4.9	4.9	4.9	4.9	5.3	4.9	4.9		97.0	94.8	94.8	94.8	94.8	94.8	94.8	94.8	94.4	94.8	95.3	95.3	95.3	95.3	92.3	94.4	95.3	24	0322J
25	0.4	1.7	4.4	1.7	1.3	3.1	1.7	1.7	1.7	2.6	2.6	1.7	1.7	1.7	1.7	1.7	1.7	1.7	1.7	1.7	2.2	1.7	1.7	3.1		97.9	97.9	97.9	97.9	97.9	97.9	97.9	97.4	97.9	98.3	98.3	98.3	98.3	94.4	97.4	98.3	25	0322A
26	1.7	3.1	4.9	3.1	2.6	3.5	3.1	3.1	3.1	4.0	4.0	3.1	3.1	3.1	3.1	3.1	0.4	0.4	3.1	3.1	0.9	3.1	3.1	5.3	2.2		100.0	100.0	100.0	100.0	100.0	100.0	98.7	100.0	99.6	99.6	97.0	99.6	93.1	96.6	99.6	26	0307F
27	1.7	3.1	4.9	3.1	2.6	3.5	3.1	3.1	3.1	4.0	4.0	3.1	3.1	3.1	3.1	3.1	0.4	0.4	3.1	3.1	0.9	3.1	3.1	5.3	2.2	0.0		100.0	100.0	100.0	100.0	100.0	98.7	100.0	99.6	99.6	97.0	99.6	93.1	96.6	99.6	27	0306W
28	1.7	3.1	4.9	3.1	2.6	3.5	3.1	3.1	3.1	4.0	4.0	3.1	3.1	3.1	3.1	3.1	0.4	0.4	3.1	3.1	0.9	3.1	3.1	5.3	2.2	0.0	0.0		100.0	100.0	100.0	100.0	98.7	100.0	99.6	99.6	97.0	99.6	93.1	96.6	99.6	28	0306L
29	1.7	3.1	4.9	3.1	2.6	3.5	3.1	3.1	3.1	4.0	4.0	3.1	3.1	3.1	3.1	3.1	0.4	0.4	3.1	3.1	0.9	3.1	3.1	5.3	2.2	0.0	0.0	0.0		100.0	100.0	100.0	98.7	100.0	99.6	99.6	97.0	99.6	93.1	96.6	99.6	29	0305O
30	1.7	3.1	4.9	3.1	2.6	3.5	3.1	3.1	3.1	4.0	4.0	3.1	3.1	3.1	3.1	3.1	0.4	0.4	3.1	3.1	0.9	3.1	3.1	5.3	2.2	0.0	0.0	0.0	0.0		100.0	100.0	98.7	100.0	99.6	99.6	97.0	99.6	93.1	96.6	99.6	30	0304R
31	1.7	3.1	4.9	3.1	2.6	3.5	3.1	3.1	3.1	4.0	4.0	3.1	3.1	3.1	3.1	3.1	0.4	0.4	3.1	3.1	0.9	3.1	3.1	5.3	2.2	0.0	0.0	0.0	0.0	0.0		100.0	98.7	100.0	99.6	99.6	97.0	99.6	93.1	96.6	99.6	31	0304J2
32	1.7	3.1	4.9	3.1	2.6	3.5	3.1	3.1	3.1	4.0	4.0	3.1	3.1	3.1	3.1	3.1	0.4	0.4	3.1	3.1	0.9	3.1	3.1	5.3	2.2	0.0	0.0	0.0	0.0	0.0	0.0		98.7	100.0	99.6	99.6	97.0	99.6	93.1	96.6	99.6	32	0304J1
33	2.2	3.5	5.3	3.5	3.1	4.0	3.5	3.5	3.5	4.4	4.4	3.5	3.5	3.5	3.5	3.5	0.9	0.9	3.5	3.5	1.3	3.5	3.5	5.8	2.6	1.3	1.3	1.3	1.3	1.3	1.3	1.3		98.7	99.1	99.1	96.6	99.1	92.7	96.1	99.1	33	0304H
34	1.7	3.1	4.9	3.1	2.6	3.5	3.1	3.1	3.1	4.0	4.0	3.1	3.1	3.1	3.1	3.1	0.4	0.4	3.1	3.1	0.9	3.1	3.1	5.3	2.2	0.0	0.0	0.0	0.0	0.0	0.0	0.0	1.3		99.6	99.6	97.0	99.6	93.1	96.6	99.6	34	0304E
35	1.3	2.6	4.4	2.6	2.2	3.1	2.6	2.6	2.6	3.5	3.5	2.6	2.6	2.6	2.6	2.6	0.0	0.0	2.6	2.6	0.4	2.6	2.6	4.9	1.7	0.4	0.4	0.4	0.4	0.4	0.4	0.4	0.9	0.4		100.0	97.4	100.0	93.6	97.0	100.0	35	0125W
36	1.3	2.6	4.4	2.6	2.2	3.1	2.6	2.6	2.6	3.5	3.5	2.6	2.6	2.6	2.6	2.6	0.0	0.0	2.6	2.6	0.4	2.6	2.6	4.9	1.7	0.4	0.4	0.4	0.4	0.4	0.4	0.4	0.9	0.4	0.0		97.4	100.0	93.6	97.0	100.0	36	0125Q1
37	1.3	0.9	4.4	0.9	0.4	3.1	0.0	0.0	0.0	1.7	1.7	0.9	0.9	0.0	0.0	0.0	2.6	2.6	0.9	0.0	3.1	0.9	0.0	4.9	1.7	3.1	3.1	3.1	3.1	3.1	3.1	3.1	3.5	3.1	2.6	2.6		97.4	94.8	98.3	97.4	37	0119S
38	1.3	2.6	4.4	2.6	2.2	3.1	2.6	2.6	2.6	3.5	3.5	2.6	2.6	2.6	2.6	2.6	0.0	0.0	2.6	2.6	0.4	2.6	2.6	4.9	1.7	0.4	0.4	0.4	0.4	0.4	0.4	0.4	0.9	0.4	0.0	0.0	2.6		93.6	97.0	100.0	38	0117R
39	5.3	5.3	8.7	4.9	4.9	7.2	5.3	5.3	5.3	5.8	5.8	4.9	4.9	5.3	5.3	5.3	6.7	6.7	4.9	5.3	7.2	5.3	5.3	8.2	5.8	7.2	7.2	7.2	7.2	7.2	7.2	7.2	7.7	7.2	6.7	6.7	5.3	6.7		94.4	93.6	39	0117G
40	2.2	1.7	4.4	0.9	1.3	3.1	1.7	1.7	1.7	1.7	1.7	0.9	0.9	1.7	1.7	1.7	3.1	3.1	0.9	1.7	3.5	1.7	1.7	5.8	2.6	3.5	3.5	3.5	3.5	3.5	3.5	3.5	4.0	3.5	3.1	3.1	1.7	3.1	5.8		97.0	40	0116D1
41	1.3	2.6	4.4	2.6	2.2	3.1	2.6	2.6	2.6	3.5	3.5	2.6	2.6	2.6	2.6	2.6	0.0	0.0	2.6	2.6	0.4	2.6	2.6	4.9	1.7	0.4	0.4	0.4	0.4	0.4	0.4	0.4	0.9	0.4	0.0	0.0	2.6	0.0	6.7	3.1		41	0116C2
	1	2	3	4	5	6	7	8	9	10	11	12	13	14	15	16	17	18	19	20	21	22	23	24	25	26	27	28	29	30	31	32	33	34	35	36	37	38	39	40	41		

Supplementary Figure S6. Amino acid homology analysis of the PCV3 ORF2 gene.

	1	2	3	4	5	6	7	8	9	10	11	12	13	14	15	16	17	18	19	20	21	22	23	24	25	26	27	28	29	30	31	32	33	34	35	36	37	38	39	40	41	42	43	44		
1		98.9	97.9	98.9	99.5	99.5	98.9	99.5	98.9	99.5	98.4	98.4	98.4	98.4	98.4	98.4	97.9	97.9	98.4	98.4	98.4	98.4	98.4	98.4	98.4	98.4	98.4	98.4	98.4	98.4	98.4	98.9	98.9	97.9	97.9	97.9	98.4	99.5	98.9	97.4	98.4	98.9	98.4	98.4	1	MG934295-PCV3a
2	1.1		98.9	100.0	99.5	99.5	100.0	99.5	100.0	99.5	98.4	98.4	98.4	98.4	98.4	98.4	97.9	97.9	98.4	98.4	98.4	98.4	98.4	98.4	98.4	98.4	98.4	98.4	98.4	98.4	98.4	98.9	98.9	97.9	97.9	97.9	98.4	99.5	98.9	97.4	98.4	98.9	98.4	98.4	2	MG770387-PCV3a
3	2.1	1.1		98.9	98.4	98.4	98.9	98.4	98.9	98.4	97.4	97.4	97.4	97.4	97.4	97.4	96.8	96.8	97.4	97.4	97.4	97.4	97.4	97.4	97.4	97.4	97.4	97.4	97.4	97.4	97.4	97.9	97.9	96.8	96.8	96.8	97.4	98.4	97.9	96.3	97.4	97.9	97.4	97.4	3	MG770384-PCV3a
4	1.1	0.0	1.1		99.5	99.5	100.0	99.5	100.0	99.5	98.4	98.4	98.4	98.4	98.4	98.4	97.9	97.9	98.4	98.4	98.4	98.4	98.4	98.4	98.4	98.4	98.4	98.4	98.4	98.4	98.4	98.9	98.9	97.9	97.9	97.9	98.4	99.5	98.9	97.4	98.4	98.9	98.4	98.4	4	KX778720-PCV3a
5	0.5	0.5	1.6	0.5		100.0	99.5	100.0	99.5	100.0	98.9	98.9	98.9	98.9	98.9	98.9	98.4	98.4	98.9	98.9	98.9	98.9	98.9	98.9	98.9	98.9	98.9	98.9	98.9	98.9	98.9	99.5	99.5	98.4	98.4	98.4	98.9	100.0	99.5	97.9	98.9	99.5	98.9	98.9	5	KY354054-PCV3a
6	0.5	0.5	1.6	0.5	0.0		99.5	100.0	99.5	100.0	98.9	98.9	98.9	98.9	98.9	98.9	98.4	98.4	98.9	98.9	98.9	98.9	98.9	98.9	98.9	98.9	98.9	98.9	98.9	98.9	98.9	99.5	99.5	98.4	98.4	98.4	98.9	100.0	99.5	97.9	98.9	99.5	98.9	98.9	6	MH603546-PCV3a
7	1.1	0.0	1.1	0.0	0.5	0.5		99.5	100.0	99.5	98.4	98.4	98.4	98.4	98.4	98.4	97.9	97.9	98.4	98.4	98.4	98.4	98.4	98.4	98.4	98.4	98.4	98.4	98.4	98.4	98.4	98.9	98.9	97.9	97.9	97.9	98.4	99.5	98.9	97.4	98.4	98.9	98.4	98.4	7	MK033207-PCV3a
8	0.5	0.5	1.6	0.5	0.0	0.0	0.5		99.5	100.0	98.9	98.9	98.9	98.9	98.9	98.9	98.4	98.4	98.9	98.9	98.9	98.9	98.9	98.9	98.9	98.9	98.9	98.9	98.9	98.9	98.9	99.5	99.5	98.4	98.4	98.4	98.9	100.0	99.5	97.9	98.9	99.5	98.9	98.9	8	KY354068-PCV3a
9	1.1	0.0	1.1	0.0	0.5	0.5	0.0	0.5		99.5	98.4	98.4	98.4	98.4	98.4	98.4	97.9	97.9	98.4	98.4	98.4	98.4	98.4	98.4	98.4	98.4	98.4	98.4	98.4	98.4	98.4	98.9	98.9	97.9	97.9	97.9	98.4	99.5	98.9	97.4	98.4	98.9	98.4	98.4	9	MH277111-PCV3a
10	0.5	0.5	1.6	0.5	0.0	0.0	0.5	0.0	0.5		98.9	98.9	98.9	98.9	98.9	98.9	98.4	98.4	98.9	98.9	98.9	98.9	98.9	98.9	98.9	98.9	98.9	98.9	98.9	98.9	98.9	99.5	99.5	98.4	98.4	98.4	98.9	100.0	99.5	97.9	98.9	99.5	98.9	98.9	10	MF631804-PCV3a
11	1.6	1.6	2.7	1.6	1.1	1.1	1.6	1.1	1.6	1.1		100.0	100.0	100.0	100.0	100.0	99.5	99.5	100.0	100.0	98.9	98.9	98.9	98.9	98.9	98.9	98.9	98.9	98.9	98.9	98.9	98.4	98.4	98.4	98.4	98.4	98.9	98.9	98.4	96.8	97.9	98.4	98.9	97.9	11	MF589107-PCV3b
12	1.6	1.6	2.7	1.6	1.1	1.1	1.6	1.1	1.6	1.1	0.0		100.0	100.0	100.0	100.0	99.5	99.5	100.0	100.0	98.9	98.9	98.9	98.9	98.9	98.9	98.9	98.9	98.9	98.9	98.9	98.4	98.4	98.4	98.4	98.4	98.9	98.9	98.4	96.8	97.9	98.4	98.9	97.9	12	MF589133-PCV3b
13	1.6	1.6	2.7	1.6	1.1	1.1	1.6	1.1	1.6	1.1	0.0	0.0		100.0	100.0	100.0	99.5	99.5	100.0	100.0	98.9	98.9	98.9	98.9	98.9	98.9	98.9	98.9	98.9	98.9	98.9	98.4	98.4	98.4	98.4	98.4	98.9	98.9	98.4	96.8	97.9	98.4	98.9	97.9	13	MG868943-PCV3b
14	1.6	1.6	2.7	1.6	1.1	1.1	1.6	1.1	1.6	1.1	0.0	0.0	0.0		100.0	100.0	99.5	99.5	100.0	100.0	98.9	98.9	98.9	98.9	98.9	98.9	98.9	98.9	98.9	98.9	98.9	98.4	98.4	98.4	98.4	98.4	98.9	98.9	98.4	96.8	97.9	98.4	98.9	97.9	14	MG934298-PCV3b
15	1.6	1.6	2.7	1.6	1.1	1.1	1.6	1.1	1.6	1.1	0.0	0.0	0.0	0.0		100.0	99.5	99.5	100.0	100.0	98.9	98.9	98.9	98.9	98.9	98.9	98.9	98.9	98.9	98.9	98.9	98.4	98.4	98.4	98.4	98.4	98.9	98.9	98.4	96.8	97.9	98.4	98.9	97.9	15	MK178284-PCV3b
16	1.6	1.6	2.7	1.6	1.1	1.1	1.6	1.1	1.6	1.1	0.0	0.0	0.0	0.0	0.0		99.5	99.5	100.0	100.0	98.9	98.9	98.9	98.9	98.9	98.9	98.9	98.9	98.9	98.9	98.9	98.4	98.4	98.4	98.4	98.4	98.9	98.9	98.4	96.8	97.9	98.4	98.9	97.9	16	MK178295-PCV3b
17	2.1	2.1	3.2	2.1	1.6	1.6	2.1	1.6	2.1	1.6	0.5	0.5	0.5	0.5	0.5	0.5		98.9	99.5	99.5	98.4	98.4	98.4	98.4	98.4	98.4	98.4	98.4	98.4	98.4	98.4	97.9	97.9	97.9	97.9	97.9	98.4	98.4	97.9	96.3	97.4	97.9	98.4	97.4	17	MK185651-PCV3b
18	2.1	2.1	3.2	2.1	1.6	1.6	2.1	1.6	2.1	1.6	0.5	0.5	0.5	0.5	0.5	0.5	1.1		99.5	99.5	98.4	98.4	98.4	98.4	98.4	98.4	98.4	98.4	98.4	98.4	98.4	97.9	97.9	97.9	97.9	97.9	98.4	98.4	97.9	96.3	97.4	97.9	98.4	97.4	18	MK347415-PCV3b
19	1.6	1.6	2.7	1.6	1.1	1.1	1.6	1.1	1.6	1.1	0.0	0.0	0.0	0.0	0.0	0.0	0.5	0.5		100.0	98.9	98.9	98.9	98.9	98.9	98.9	98.9	98.9	98.9	98.9	98.9	98.4	98.4	98.4	98.4	98.4	98.9	98.9	98.4	96.8	97.9	98.4	98.9	97.9	19	MG868940-PCV3b
20	1.6	1.6	2.7	1.6	1.1	1.1	1.6	1.1	1.6	1.1	0.0	0.0	0.0	0.0	0.0	0.0	0.5	0.5	0.0		98.9	98.9	98.9	98.9	98.9	98.9	98.9	98.9	98.9	98.9	98.9	98.4	98.4	98.4	98.4	98.4	98.9	98.9	98.4	96.8	97.9	98.4	98.9	97.9	20	MN605934-PCV3b
21	1.6	1.6	2.7	1.6	1.1	1.1	1.6	1.1	1.6	1.1	1.1	1.1	1.1	1.1	1.1	1.1	1.6	1.6	1.1	1.1		100.0	100.0	100.0	100.0	100.0	100.0	100.0	100.0	100.0	100.0	98.4	98.4	99.5	99.5	99.5	100.0	98.9	98.4	96.8	97.9	98.4	100.0	97.9	21	KX966193-PCV3c
22	1.6	1.6	2.7	1.6	1.1	1.1	1.6	1.1	1.6	1.1	1.1	1.1	1.1	1.1	1.1	1.1	1.6	1.6	1.1	1.1	0.0		100.0	100.0	100.0	100.0	100.0	100.0	100.0	100.0	100.0	98.4	98.4	99.5	99.5	99.5	100.0	98.9	98.4	96.8	97.9	98.4	100.0	97.9	22	KY996343-PCV3c
23	1.6	1.6	2.7	1.6	1.1	1.1	1.6	1.1	1.6	1.1	1.1	1.1	1.1	1.1	1.1	1.1	1.6	1.6	1.1	1.1	0.0	0.0		100.0	100.0	100.0	100.0	100.0	100.0	100.0	100.0	98.4	98.4	99.5	99.5	99.5	100.0	98.9	98.4	96.8	97.9	98.4	100.0	97.9	23	KY354050-PCV3c
24	1.6	1.6	2.7	1.6	1.1	1.1	1.6	1.1	1.6	1.1	1.1	1.1	1.1	1.1	1.1	1.1	1.6	1.6	1.1	1.1	0.0	0.0	0.0		100.0	100.0	100.0	100.0	100.0	100.0	100.0	98.4	98.4	99.5	99.5	99.5	100.0	98.9	98.4	96.8	97.9	98.4	100.0	97.9	24	MF162298-PCV3c
25	1.6	1.6	2.7	1.6	1.1	1.1	1.6	1.1	1.6	1.1	1.1	1.1	1.1	1.1	1.1	1.1	1.6	1.6	1.1	1.1	0.0	0.0	0.0	0.0		100.0	100.0	100.0	100.0	100.0	100.0	98.4	98.4	99.5	99.5	99.5	100.0	98.9	98.4	96.8	97.9	98.4	100.0	97.9	25	MF589110-PCV3c
26	1.6	1.6	2.7	1.6	1.1	1.1	1.6	1.1	1.6	1.1	1.1	1.1	1.1	1.1	1.1	1.1	1.6	1.6	1.1	1.1	0.0	0.0	0.0	0.0	0.0		100.0	100.0	100.0	100.0	100.0	98.4	98.4	99.5	99.5	99.5	100.0	98.9	98.4	96.8	97.9	98.4	100.0	97.9	26	MF769811-PCV3c
27	1.6	1.6	2.7	1.6	1.1	1.1	1.6	1.1	1.6	1.1	1.1	1.1	1.1	1.1	1.1	1.1	1.6	1.6	1.1	1.1	0.0	0.0	0.0	0.0	0.0	0.0		100.0	100.0	100.0	100.0	98.4	98.4	99.5	99.5	99.5	100.0	98.9	98.4	96.8	97.9	98.4	100.0	97.9	27	MG870097-PCV3c
28	1.6	1.6	2.7	1.6	1.1	1.1	1.6	1.1	1.6	1.1	1.1	1.1	1.1	1.1	1.1	1.1	1.6	1.6	1.1	1.1	0.0	0.0	0.0	0.0	0.0	0.0	0.0		100.0	100.0	100.0	98.4	98.4	99.5	99.5	99.5	100.0	98.9	98.4	96.8	97.9	98.4	100.0	97.9	28	MH277114-PCV3c
29	1.6	1.6	2.7	1.6	1.1	1.1	1.6	1.1	1.6	1.1	1.1	1.1	1.1	1.1	1.1	1.1	1.6	1.6	1.1	1.1	0.0	0.0	0.0	0.0	0.0	0.0	0.0	0.0		100.0	100.0	98.4	98.4	99.5	99.5	99.5	100.0	98.9	98.4	96.8	97.9	98.4	100.0	97.9	29	MW167067-PCV3c
30	1.6	1.6	2.7	1.6	1.1	1.1	1.6	1.1	1.6	1.1	1.1	1.1	1.1	1.1	1.1	1.1	1.6	1.6	1.1	1.1	0.0	0.0	0.0	0.0	0.0	0.0	0.0	0.0	0.0		100.0	98.4	98.4	99.5	99.5	99.5	100.0	98.9	98.4	96.8	97.9	98.4	100.0	97.9	30	MN698814-PCV3c
31	1.6	1.6	2.7	1.6	1.1	1.1	1.6	1.1	1.6	1.1	1.1	1.1	1.1	1.1	1.1	1.1	1.6	1.6	1.1	1.1	0.0	0.0	0.0	0.0	0.0	0.0	0.0	0.0	0.0	0.0		98.4	98.4	99.5	99.5	99.5	100.0	98.9	98.4	96.8	97.9	98.4	100.0	97.9	31	0702H
32	1.1	1.1	2.1	1.1	0.5	0.5	1.1	0.5	1.1	0.5	1.6	1.6	1.6	1.6	1.6	1.6	2.1	2.1	1.6	1.6	1.6	1.6	1.6	1.6	1.6	1.6	1.6	1.6	1.6	1.6	1.6		98.9	97.9	97.9	97.9	98.4	99.5	100.0	98.4	98.4	100.0	98.4	98.4	32	06186
33	1.1	1.1	2.1	1.1	0.5	0.5	1.1	0.5	1.1	0.5	1.6	1.6	1.6	1.6	1.6	1.6	2.1	2.1	1.6	1.6	1.6	1.6	1.6	1.6	1.6	1.6	1.6	1.6	1.6	1.6	1.6	1.1		97.9	97.9	97.9	98.4	99.5	98.9	97.4	98.4	98.9	98.4	98.4	33	0618I
34	2.1	2.1	3.2	2.1	1.6	1.6	2.1	1.6	2.1	1.6	1.6	1.6	1.6	1.6	1.6	1.6	2.1	2.1	1.6	1.6	0.5	0.5	0.5	0.5	0.5	0.5	0.5	0.5	0.5	0.5	0.5	2.1	2.1		98.9	98.9	99.5	98.4	97.9	96.3	97.4	97.9	99.5	97.4	34	0604I
35	2.1	2.1	3.2	2.1	1.6	1.6	2.1	1.6	2.1	1.6	1.6	1.6	1.6	1.6	1.6	1.6	2.1	2.1	1.6	1.6	0.5	0.5	0.5	0.5	0.5	0.5	0.5	0.5	0.5	0.5	0.5	2.1	2.1	1.1		98.9	99.5	98.4	97.9	96.3	97.4	97.9	99.5	97.4	35	0604O
36	2.1	2.1	3.2	2.1	1.6	1.6	2.1	1.6	2.1	1.6	1.6	1.6	1.6	1.6	1.6	1.6	2.1	2.1	1.6	1.6	0.5	0.5	0.5	0.5	0.5	0.5	0.5	0.5	0.5	0.5	0.5	2.1	2.1	1.1	1.1		99.5	98.4	97.9	96.3	97.4	97.9	99.5	97.4	36	0424Q
37	1.6	1.6	2.7	1.6	1.1	1.1	1.6	1.1	1.6	1.1	1.1	1.1	1.1	1.1	1.1	1.1	1.6	1.6	1.1	1.1	0.0	0.0	0.0	0.0	0.0	0.0	0.0	0.0	0.0	0.0	0.0	1.6	1.6	0.5	0.5	0.5		98.9	98.4	96.8	97.9	98.4	100.0	97.9	37	0402H
38	0.5	0.5	1.6	0.5	0.0	0.0	0.5	0.0	0.5	0.0	1.1	1.1	1.1	1.1	1.1	1.1	1.6	1.6	1.1	1.1	1.1	1.1	1.1	1.1	1.1	1.1	1.1	1.1	1.1	1.1	1.1	0.5	0.5	1.6	1.6	1.6	1.1		99.5	97.9	98.9	99.5	98.9	98.9	38	0401H1
39	1.1	1.1	2.1	1.1	0.5	0.5	1.1	0.5	1.1	0.5	1.6	1.6	1.6	1.6	1.6	1.6	2.1	2.1	1.6	1.6	1.6	1.6	1.6	1.6	1.6	1.6	1.6	1.6	1.6	1.6	1.6	0.0	1.1	2.1	2.1	2.1	1.6	0.5		98.4	98.4	100.0	98.4	98.4	39	0116F2
40	2.7	2.7	3.8	2.7	2.1	2.1	2.7	2.1	2.7	2.1	3.2	3.2	3.2	3.2	3.2	3.2	3.8	3.8	3.2	3.2	3.2	3.2	3.2	3.2	3.2	3.2	3.2	3.2	3.2	3.2	3.2	1.6	2.7	3.8	3.8	3.8	3.2	2.1	1.6		96.8	98.4	96.8	96.8	40	0328M
41	1.6	1.6	2.7	1.6	1.1	1.1	1.6	1.1	1.6	1.1	2.1	2.1	2.1	2.1	2.1	2.1	2.7	2.7	2.1	2.1	2.1	2.1	2.1	2.1	2.1	2.1	2.1	2.1	2.1	2.1	2.1	1.6	1.6	2.7	2.7	2.7	2.1	1.1	1.6	3.2		98.4	97.9	98.9	41	0322P
42	1.1	1.1	2.1	1.1	0.5	0.5	1.1	0.5	1.1	0.5	1.6	1.6	1.6	1.6	1.6	1.6	2.1	2.1	1.6	1.6	1.6	1.6	1.6	1.6	1.6	1.6	1.6	1.6	1.6	1.6	1.6	0.0	1.1	2.1	2.1	2.1	1.6	0.5	0.0	1.6	1.6		98.4	98.4	42	0125Q1
43	1.6	1.6	2.7	1.6	1.1	1.1	1.6	1.1	1.6	1.1	1.1	1.1	1.1	1.1	1.1	1.1	1.6	1.6	1.1	1.1	0.0	0.0	0.0	0.0	0.0	0.0	0.0	0.0	0.0	0.0	0.0	1.6	1.6	0.5	0.5	0.5	0.0	1.1	1.6	3.2	2.1	1.6		97.9	43	0111R
44	1.6	1.6	2.7	1.6	1.1	1.1	1.6	1.1	1.6	1.1	2.1	2.1	2.1	2.1	2.1	2.1	2.7	2.7	2.1	2.1	2.1	2.1	2.1	2.1	2.1	2.1	2.1	2.1	2.1	2.1	2.1	1.6	1.6	2.7	2.7	2.7	2.1	1.1	1.6	3.2	1.1	1.6	2.1		44	121283R
	1	2	3	4	5	6	7	8	9	10	11	12	13	14	15	16	17	18	19	20	21	22	23	24	25	26	27	28	29	30	31	32	33	34	35	36	37	38	39	40	41	42	43	44		
